# Quercetin suppresses the progression of HBV-associated hepatocellular carcinoma by modulating the EGFR signaling pathway

**DOI:** 10.1371/journal.pone.0350584

**Published:** 2026-06-12

**Authors:** Zhijuan Wang, Jinzhen Huang, Danping Huang, Rui An, Guangdong Tong, Weiqu Yuan, Mianmian Liao, Qiusheng Zhong

**Affiliations:** 1 Dongguan Key Laboratory of Traditional Chinese Medicine Hepatology, the Affiliated Dongguan Songshan Lake Central Hospital, Guangdong Medical University, Dongguan, China (Primary Supporting Institution); 2 Guangdong Pharmaceutical University, No. 280 East Huan Road, University City, Panyu District, Guangdong Province, China; 3 Guangzhou University of Chinese Medicine Shenzhen Hospital (Futian), No. 1, Fuhua Road, Futian District, Shenzhen City, Guangdong Province, China; 4 Department of Acupuncture, Shenzhen Traditional Chinese Medicine Hospital, The Fourth Clinical Medical College of Guangzhou University of Chinese Medicine, Shenzhen, Guangdong Province, China; Universita degli Studi della Campania Luigi Vanvitelli, ITALY

## Abstract

**Background:**

Quercetin, a bioactive flavonoid compound widely present in medicinal and edible plants, has demonstrated therapeutic potential against hepatocellular carcinoma (HCC). However, its specific mechanism in hepatitis B virus-associated hepatocellular carcinoma (HBV-HCC) remains unclear. This study aims to systematically elucidate the efficacy and molecular mechanisms of quercetin against HBV-HCC.

**Methods:**

Integrated in vitro and in vivo experimental models were employed. The inhibitory effects of quercetin on HCC cell viability, proliferation, clonogenicity, and migration were assessed through CCK-8, EdU, colony formation, and scratch assays. Network pharmacology and molecular docking were integrated to identify potential targets of quercetin within HCC. Lentiviral transfection was used to construct HCC cell lines overexpressing HBx and EGFR, with key signaling pathways verified via Western blot. Additionally, a xenograft mouse model was established, and the EGFR inhibitor osimertinib was combined to evaluate quercetin’s therapeutic efficacy and underlying mechanisms in HBV-HCC.

**Results:**

Through an extensive analysis of the target interaction network analysis of quercetin in HCC, this study identified and prioritized 29 potential therapeutic targets, with the EGFR recognized as the principal target molecule. The results of molecular docking experiments indicated that both EGFR and GSK3β exhibited good binding affinity. Subsequent in vitro studies revealed that quercetin substantially suppresses the growth and migration of HBV-HCC cells. It achieves this by dose-dependently suppressing EGFR, thereby attenuating the signaling of the downstream PI3K/AKT/GSK3β axis and concurrently reversing the epithelial-mesenchymal transition (EMT) process. In vivo investigations, complemented by control studies using EGFR inhibitors, further validate that quercetin exerts its anti-tumor effects against HBV-HCC through specific targeting of EGFR and the suppression of the EMT program.

**Conclusion:**

This research validates the therapeutic effectiveness of quercetin in inhibiting HBV-HCC and elucidates the molecular mechanisms responsible for its action. Mechanistically, quercetin inhibits the PI3K/AKT/GSK3β signaling axis by targeting EGFR, thereby reversing the EMT process and ultimately impeding HBV-HCC progression. These critical outcomes provide a novel theoretical foundation for the targeted therapy of HBV-HCC using quercetin.

## 1. Introduction

Globally, cancer remains a leading challenge for public health systems, posing a significant threat to global health [1,2]. Among various malignancies, hepatocellular carcinoma (HCC) is particularly notable for its high incidence and mortality. Epidemiological data reveal that nearly 900,000 new cases of HCC are diagnosed worldwide annually, with approximately 750,000 deaths attributed to the disease each year [3,4]. Persistent hepatitis B virus (HBV) infection represents a primary etiological factor of HCC development, epidemiologically accounting for 55–80% of global HCC cases [5]. Due to the insidious onset of symptoms, early-stage HBV-HCC is often diagnosed at intermediate or advanced stages, thereby precluding many patients from curative surgical interventions [6]. Although significant advancements have been made in therapeutic modalities—including surgical resection, microwave ablation, chemotherapy, immunotherapy, and targeted therapy—these approaches are constrained by considerable limitations [5,6]. These include technical complexity, high post-treatment recurrence rates, suboptimal long-term survival, and the development of treatment resistance [7]. Therefore, the development of innovative therapeutic strategies is urgently needed to overcome the clinical challenges of HBV-HCC and reduce its global disease burden.

EGFR, a transmembrane receptor tyrosine kinase of the ERBB family, has a glycosylation-dependent structure that is critical for cellular regulation [8]. Upon binding to its specific ligands—such as epidermal growth factor (EGF), transforming growth factor-alpha (TGF-α), and amphiregulin—EGFR undergoes homo-or heterodimerization, which triggers autophosphorylation of its intracellular domain and subsequently activates key downstream cascades, including the PI3K/AKT/mTOR, STAT, and RAS/MEK/ERK pathways [9]. EGFR is frequently overexpressed in numerous malignancies, and its aberrant activation—along with the consequent dysregulation of downstream signaling pathways—is recognized as a hallmark driver of cancers such as non-small cell lung cancer, glioblastoma, colorectal cancer, and bladder cancer. [10–12]. In HCC, EGFR serves a pivotal function in tumor pathogenesis. Studies have demonstrated that EGFR activation augments tumor proliferation, invasion, and metastasis [13–15]. The cellular regulation of EGFR in HCC is complex, as underscored by recent discoveries of diverse regulatory mechanisms: NEK7-induced EGFR phosphorylation at Ser1070 confers resistance to lenvatinib [16]; TBC1D31 amplification sustains EGFR signaling by impairing its lysosomal degradation [17]; and RAB40C stabilizes EGFR via TRIM21 recruitment [4]. Moreover, the EGFR axis is centrally involved in therapy resistance, with the AHR-AREG-EGFR-ERK1/2 cascade and STARD4/EGFR axis both contributing to lenvatinib resistance [18,19]. These observations highlight EGFR as a critical node in HCC pathogenesis and a promising therapeutic target [20,21]. Natural products such as zedoary turmeric oil exert anti-HCC effects through EGFR pathway modulation [22], yet the specific role of EGFR in HBV-HCC remains poorly defined, prompting the current investigation.

Quercetin, a naturally occurring flavonoid, is abundant in common foods (e.g., apples, onions, berries, tea) and medicinal-edible herbs, including Astragalus (Huangqi), Scutellaria (Huangqin), Pueraria lobata (Gegen), Gardenia jasminoides (Zhizi), Lycium barbarum (Gouqi), and Glycyrrhiza uralensis (Gancao) [23]. Accumulating evidence has demonstrated that quercetin exerts significant anti-tumor effects by inducing apoptosis and suppressing the migration and proliferation of tumor cells, thereby impeding the progression of various malignancies [24–29]. Given its well-documented anti-tumor activity, the therapeutic potential of quercetin in HCC has attracted considerable research interest. Studies have shown that quercetin inhibits HCC progression through multiple mechanisms, including the induction of apoptosis, suppression of invasion and metastasis, and promotion of autophagy [30]. Nonetheless, the precise molecular mechanisms by which quercetin suppresses HBV-HCC progression remain largely unexplored, warranting further investigation.

Based on a network pharmacology and molecular docking approach, this study identified potential therapeutic targets of quercetin in HCC. Subsequent validation in both cellular and animal models consistently confirmed that quercetin markedly suppresses the propagation and migratory competence of HBV-HCC cells. Additionally, western blot analysis revealed that quercetin inhibits HBV-HCC progression primarily by targeting EGFR, thereby downregulating the PI3K/AKT/GSK3β axis and suppressing tumor cell proliferation.

## 2. Materials and methods

### 2.1. Quercetin target exploration

The molecular structure of quercetin (PubChem CID 5280343) was retrieved from the PubChem database (https://pubchem.ncbi.nlm.nih.gov/). Potential protein targets of quercetin were then predicted using the PharmMapper platform (http://www.lilab-ecust.cn/Pharmmapper/) and subsequently standardized with the UniProt database (https://www.uniprot.org/).

### 2.2. HCC disease target collection

Using ‘Hepatocellular Carcinoma’ as the search term and restricting results to Homo sapiens genes, we systematically screened three major databases: GeneCards (https://www.genecards.org/), the Therapeutic Target Database (TTD; https://db.idrblab.net/ttd/), and Online Mendelian Inheritance in Man (OMIM; https://www.omim.org/). Subsequently, the identified candidate genes were standardized using the UniProt database (https://www.uniprot.org/) by converting all target proteins to their corresponding gene symbols and confirming species as Homo sapiens, thereby ensuring standardized gene nomenclature and avoiding over-annotation of homologous proteins. For targets derived from the GeneCards database, a three-step median filtering based on Relevance score was applied to exclude low-relevance candidates before merging with targets from TTD and OMIM.

### 2.3. Protein interaction network

Common targets between quercetin and HCC were identified by mapping quercetin targets to HCC-related targets using Venny 2.1 (https://bioinfogp.cnb.csic.es/tools/venny/). These overlapping targets were then imported into the STRING database (http://string-db.org) with a minimum required interaction score of >0.4 and disconnected nodes hidden, generating protein–protein interaction (PPI) data. The node relationship information was exported in TSV format and imported into Cytoscape (v3.8.0) for visualization. Clustering analysis was subsequently performed using the MCODE plugin to identify hub proteins based on network centrality measures and clustering coefficients.

### 2.4. GO and KEGG enrichment analysis

Using the DAVID database (https://david.ncifcrf.gov/), GO and KEGG enrichment analyses were performed on the overlapping targets [31,32]. The analysis was restricted to Homo sapiens to identify significantly enriched biological processes, molecular functions, cellular components, and signaling pathways (P < 0.05).

### 2.5. Molecular docking

To evaluate the binding affinity of quercetin for potential targets, 12 core targets enriched in the EGFR pathway were selected for molecular docking. Three-dimensional protein structures were retrieved from the RCSB PDB (http://www.rcsb.org/), and the two-dimensional structure was obtained from ZINC (http://zinc.docking.org/) database. Proteins were prepared using AutoDock Tools by removing water molecules, adding polar hydrogens, and assigning Kollman charges. Molecular docking was performed with AutoDock Vina, with the grid box centered on the original ligand. Binding poses with docking energies ≤ −5.0 kcal/mol were selected as the screening criterion.

### 2.6. Cell lines and reagents

Human HCC cell lines (HepG2 and Huh-7), procured from the ATCC, were cultured in DMEM (Gibco) containing 10% FBS (Procell) and 1% penicillin/streptomycin (P/S; Gibco) at 37 ℃ with 5% CO₂. The following chemicals were used in this study: quercetin (Herbest, HR20119B1), osimertinib (MCE, AZD-9291), and lenvatinib (Selleck, S116404). All compounds were dissolved in dimethyl sulfoxide (DMSO; MP Biomedicals, YC0907) and stored at −20 ℃.

### 2.7. Lentiviral transfection

Stable cell lines were generated using lentiviral transduction as previously described [33–35]. Specifically, HepG2-HBx, Huh-7-HBx, and HepG2-HBx-EGFR cell lines were established by transducing parental HepG2 or Huh-7 cells with recombinant lentiviruses harboring either the pLVX-HBx-IRES-Puro construct (expressing HBx; GenBank: AB033559) or the pCDH-EGFR-T2A-Blast construct (encoding wild-type EGFR; UniProt: P00533). Transduction was performed in polybrene-supplemented medium (8 μg/mL) at an optimized multiplicity of infection, coupled with spinoculation (1000 × g, 32 ℃, 90 min). Following a 48-hour recovery period, stable polyclonal populations were selected with antibiotics as follows: HBx-expressing lines (HepG2-HBx, Huh-7-HBx) were maintained in 2 μg/mL puromycin for 14 days, EGFR-expressing lines were selected with 5 μg/mL blasticidin for 21 days, and the dual-modified HepG2-HBx-EGFR line was established through sequential antibiotic selection (puromycin 2 μg/mL for 14 days, followed by blasticidin 5 μg/mL for 21 days). Successful transgene expression was validated by Western blotting prior to functional assays.

### 2.8. Experimental animals

Male SPF-grade BALB/c nude mice (approximately five weeks old, 22 ± 2 g) were purchased from Guangdong Experimental Animal Center. All animal experiments were conducted in strict accordance with ethical guidelines and approved by the Animal Ethics Review Committee of the Shenzhen Institute for Drug Control (No. 20220508). Xenograft models were established by subcutaneously implanting HepG2-HBx cells (5 × 10^6^ cells in 100 μL Matrigel/PBS [1:1]) into the right flank. When tumors reached 100 ± 10 mm^3^ (typically 14 days post-implantation), mice with established tumors were randomly assigned to four groups: 1) the control group which received daily oral gavage of 0.1% DMSO, 2) the quercetin group which received daily oral gavage of 50 mg/kg quercetin solution, 3) the osimertinib group which received daily oral gavage of 15 mg/kg osimertinib solution and 4) the lenvatinib group which received daily oral gavage of 10 mg/kg lenvatinib solution. After 20 consecutive days of treatment, mice were anesthetized with 1% pentobarbital sodium via intraperitoneal injection at a dose of 30 mg/kg, and euthanized by cervical dislocation after the experiment. Tumor tissues were excised, weighed, and tumor volume was determined by applying the ellipsoid formula: Volume (mm3) = Length (mm) × [Width (mm)]^2^/ 2.

### 2.9. Cell viability assay (CCK-8)

Cell viability and growth were assessed using the CCK-8 assay (GLPBIO, GK1000). HepG2 and HepG2-HBx cells in the logarithmic growth phase were seeded into 96-well culture plates at a density of 3 × 10³ cells per well. The CCK-8 reagent was administered daily for five consecutive days. After reagent addition, the plates were subjected to 37 ℃ for 3 h. Absorbance measurements were subsequently conducted at 450 nm with a microplate reader. Growth curves were generated from the absorbance data.

To evaluate the effect of quercetin on HCC cell viability, cells in the logarithmic growth phase were seeded into 96-well plates, allowed to adhere overnight, and then treated with varying concentrations of quercetin (0, 10, 20, 30, 40, 50, 60, 70, 80, 90, 100, and 110 μM). After 24, 48, and 72 h of treatment, cell viability was assessed by measuring absorbance at 450 nm. The half-maximal inhibitory concentration (IC_50_) was determined by dose-response curve analysis.

### 2.10. Colony formation assay

Cells were seeded into 6-well plates at a density of 500 cells per well and allowed to adhere overnight. After 24 h of treatment with various concentrations of quercetin, the cells were cultured in fresh complete medium for three weeks, with medium replaced regularly. Once macroscopically visible colonies formed, the cells were rinsed with phosphate-buffered saline (PBS), fixed with 4% paraformaldehyde (Biosharp, BL539A), and then stained with a crystal violet solution (Beyotime Biotechnology, C0121). After staining, the cells were rinsed again with PBS to remove excess dye. Colony images were captured, and the number of colonies was counted for statistical analysis.

### 2.11. EdU proliferation assay

The EdU cell proliferation kit (APExBIO, K1076) was employed to evaluate the effect of quercetin on HCC cell proliferation. Cells were harvested and seeded into 96-well plates at a density of 3 × 10^5^ cells/mL in 100 μL suspension per well, followed by overnight incubation at 37 ℃ with 5% CO_2_. Upon reaching approximately 70% confluence, cells were treated with varying concentrations of quercetin for 24 h. Subsequently, cells were incubated with 20 μM EdU for 3 h at 37 ℃. After incubation, cells were fixed with 4% paraformaldehyde for 30 min and permeabilized with 0.3% Triton X-100 for 15 min. Following a 30 min incubation with Click Reaction Buffer in the dark, the nuclei were counterstained with Hoechst 33342. Fluorescence microscopy was used to capture images, and the percentage of EdU-positive cells was quantified (EdU-positive rate (%) = [Number of EdU-positive cells /Total number of Hoechst-stained cells] × 100%).

### 2.12. Transwell migration assay

Cell migratory capacity was assessed using Transwell assays (LABSELECT, 14342). Cells were suspended at a density of 8 × 10^4^ cells/mL, and 200 μL was introduced into the upper chamber. The lower chamber was filled with 500 μL of DMEM containing 30% FBS as a chemoattractant. After 24 h of incubation at 37 ℃, the chambers were fixed with 4% paraformaldehyde for 30 min and stained with crystal violet for 15 min. Migrated cells were imaged at 10 × magnification and quantified with ImageJ software.

### 2.13. Scratch assays

HCC cells were seeded into 6-well plates at a density of 5 × 10^5^ cells/well. Upon reaching 90–100% confluence, a linear scratch wound was created across the monolayer using a sterile 1 mL pipette tip. Cells were then treated with varying concentrations of quercetin for 72 h. Wound closure was monitored, and images were captured at 0, 24, 48, and 72 h post-scratch. The scratch area was quantified using ImageJ software, and cell migration ability was assessed by calculating the wound closure percentage (wound closure percentage (%) = [migrated distance / initial scratch width] × 100%).

### 2.14. Western blot

Cellular proteins were lysed using RIPA buffer (NCM, WB3100) supplemented with a protease inhibitor cocktail. Protein concentrations were determined using a BCA protein assay kit (CWBIO, CW0014S). Equal amounts of protein were denatured and separated by 8–12% SDS-PAGE, then transferred to PVDF membranes (Merck Millipore, ISEQ00010). The membranes were blocked with a blocking solution (Servicebio, CR2405137) for 1 h at room temperature and incubated with primary antibodies overnight at 4 ℃. After washing, the membranes were incubated with HRP-conjugated secondary antibodies for 1 h at room temperature. Protein bands were visualized using High-sig ECL western blotting substrate (Tanon, 180–5001) and captured with a Servicebio imaging system.

The following antibodies were used in this study: anti-β-actin (66009–1-IG, 1:3000; Proteintech), anti-VEGFR2 (26415–1-AP, 1:2000; Proteintech), anti-EGFR (18986–1-AP, 1:2000; Proteintech), anti-p-EGFR (ab40815, 1:2000; Abcam), anti-PI3K (20584–1-AP, 1:500; Proteintech), anti-p-PI3K (PC6417, 1:500; Abmart), anti-AKT (10176–2-AP, 1:3000; Proteintech), anti-p-AKT (66444–1-IG, 1:3000; Proteintech), anti-GSK3β (22104–1-AP, 1:1000; Proteintech), anti-p-GSK3β (67558–1-IG, 1:1000; Proteintech), anti-E-cadherin (20874–1-AP, 1:1000; Proteintech), anti-N-cadherin (22018–1-AP, 1:1000; Proteintech), anti-Vimentin (10366–1-AP, 1:1000; Proteintech), anti-HBx (ab2741, 1:1000; Abcam), HRP-conjugated Goat Anti-Mouse IgG (H + L) (SA00001–1, 1:5000; Proteintech), HRP-conjugated Goat Anti-Rabbit IgG (H + L) (SA00001–2, 1:5000; Proteintech). All antibodies were diluted with Universal Antibody Diluent (NCM, WB500D).

### 2.15. H&E

Tumor tissue specimens were fixed in 4% paraformaldehyde at 4 ℃ for 24 h. After fixation, the tissues were embedded in paraffin, sectioned into 5 μm thick slices using a microtome, and stained with hematoxylin and eosin.

### 2.16. IHC

After dewaxing and rehydration, paraffin-embedded tissue sections were subjected to antigen retrieval and endogenous peroxidase blocking. The sections were then incubated overnight at 4 ℃ with primary antibodies against Ki67, EGFR, N-cadherin, E-cadherin, Vimentin, and VEGFR2. After washing, the bound antibodies were visualized using a DAB chromogen kit, followed by counterstaining with hematoxylin. Stained sections were imaged using a light microscope, and three random fields per section were selected for quantitative analysis. The images were then analyzed using ImageJ software. The integrated optical density (IOD) of DAB-positive staining was measured, and the mean IOD (average density) was calculated as IOD divided by the area of the measured field, reflecting the relative expression level of each target protein. All quantitative analyses were performed under identical imaging and threshold settings to ensure consistency.

### 2.17. Statistical analyses

Statistical analysis was performed using SPSS (version 25.0) and GraphPad Prism (Version 9.5.1). All experiments were performed in triplicate, and data are presented as mean ± SD. Comparisons between two groups were analyzed using two-tailed unpaired Student’s t-test, while comparisons among multiple groups were evaluated using one-way analysis of variance (ANOVA). A value of P < 0.05 was considered statistically significant.

## 3. Result

### 3.1. Quercetin inhibits the proliferation of HCC

**Fig 1:**
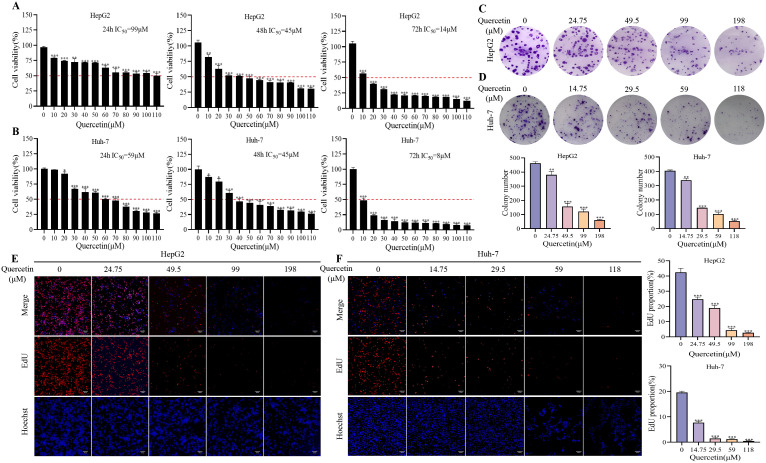
Quercetin inhibits the proliferation of HCC cells in vitro. **(A-B)** Cell viability was assessed using the CCK-8 assay after treatment with the indicated concentrations of quercetin for 24, 48, and 72 h. **(C-D)** Colony formation ability of HepG2 and Huh-7 cells was evaluated following quercetin treatment at the indicated concentrations for 24 h. **(E-F)** Cell proliferation was assessed using the EdU incorporation assay after quercetin treatment at the indicated concentrations for 24 h. All experiments were performed in triplicate (n = 3). Data are presented as mean ± SD. Note: ^*^P < 0.05, ^**^P < 0.01, ^***^P < 0.001 vs. control group.

To investigate the impact of quercetin on HCC cell proliferation, we performed CCK-8, colony formation, and EdU incorporation assays. CCK-8 assays demonstrated that quercetin significantly inhibited the growth of HepG2 and Huh-7 cells in a dose- and time-dependent manner. The IC_50_ values were calculated to be 99 μM, 45 μM, and 14 μM for HepG2 cells, and 59 μM, 45 μM, and 8 μM for Huh-7 cells at 24, 48, and 72 h, respectively (Fig 1A, B). In line with these findings, colony formation assays revealed that quercetin treatment led to a dose-dependent reduction in the number and size of colonies in both cell lines (Fig 1C, D). Moreover, EdU incorporation assays indicated that quercetin decreased the EdU-positive rate in HepG2 and Huh-7 cells, indicating that quercetin could restrain the proliferation of HCC (Fig 1E, F). These data establish that quercetin potently inhibits HCC cell proliferation.

### 3.2. Identification of Quercetin’s potential therapeutic targets in HCC

**Fig 2:**
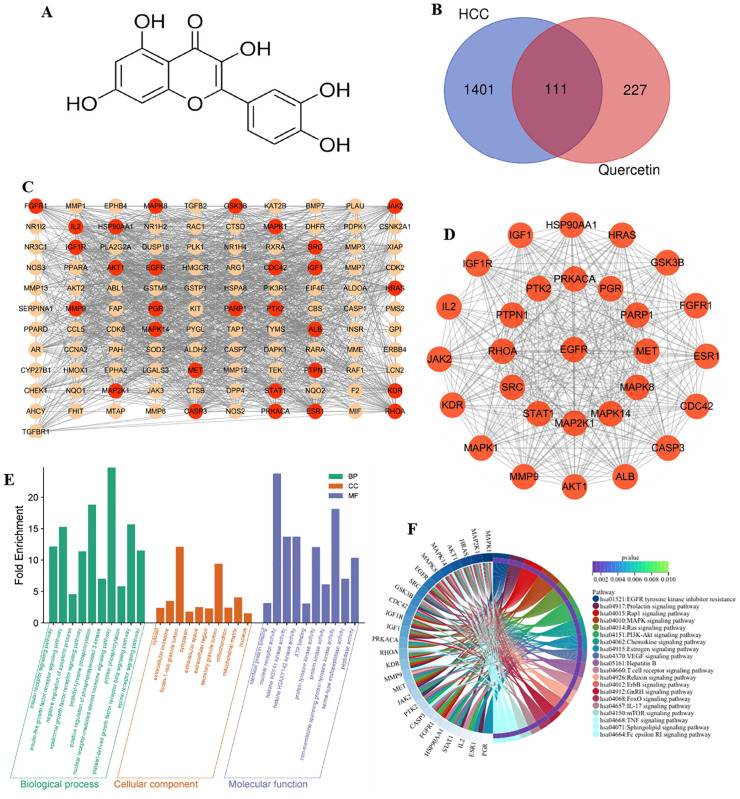
Screening of potential targets for quercetin in the prevention and treatment of HCC. **(A)** Molecular structure of quercetin. **(B)** Venn diagram illustrating the screening of HCC-quercetin targets. **(C)** PPI network of the shared targets between quercetin and HCC. **(D)** Interaction network of core targets for quercetin in HCC prevention and treatment. **(E)** A histogram representing GO enrichment analysis. **(F)** KEGG (www.kegg.jp/kegg/kegg1.html) pathway enrichment analysis.

To elucidate the molecular targets and underlying mechanisms of quercetin in HCC, we employed a network pharmacology approach. A total of 227 potential quercetin targets were identified after standardization against the UniProt database. HCC-related targets were retrieved from the GeneCards, TTD, and OMIM databases, resulting in 1,401 targets. Venn diagram analysis revealed 111 overlapping targets between quercetin and HCC (Fig 2B). These common targets were then imported into the STRING database to construct a protein-protein interaction (PPI) network. After removing isolated nodes in Cytoscape, MCODE cluster analysis identified the most significant functional module (Cluster 1), consisting of 29 core targets (Fig 2C, D).

To explore the biological functions of these core targets, GO and KEGG enrichment analysis were performed. GO enrichment analysis (P < 0.05) identified 72 biological processes (including insulin receptor and growth factor receptor signaling pathways), 70 molecular functions (such as histone kinase activity and nuclear receptor activity), and 57 cellular components (including cytoplasmic vesicles and extracellular exosomes) (Fig 2E). KEGG pathway analysis revealed 126 significantly enriched pathways (P < 0.05), among which the EGFR tyrosine kinase inhibitor resistance pathway (hsa01521) showed the strongest association with HCC pathogenesis (Fig 2F). These findings suggest that quercetin may suppress HCC by modulating the EGFR pathway.

**Fig 3:**
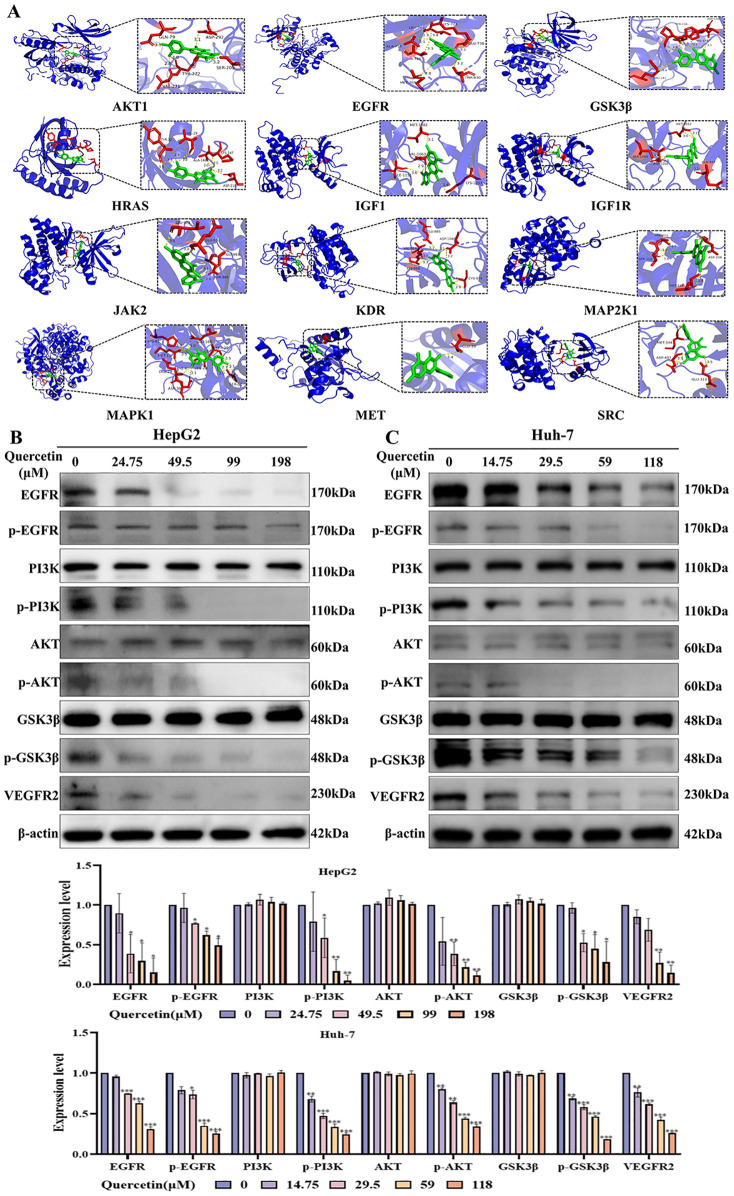
Molecular docking and investigation of the mechanism of quercetin in inhibiting HCC. **(A)** Molecular docking results between quercetin and the 12 core target proteins. **(B-C)** Western blot analysis was performed to evaluate the expression of EGFR and downstream signaling proteins (PI3K, AKT, GSK3β) in HCC cells after treatment with the indicated concentrations of quercetin for 24 h.. All experiments were performed in triplicate (n = 3). Data are presented as mean ± SD. Note: ^*^P < 0.05, ^**^P < 0.01, ^***^P < 0.001 vs. control group.

Molecular docking was performed to evaluate the binding affinities of quercetin with 12 key protein targets. As shown in Fig 3A, all 12 targets exhibited binding energies ≤ −5.0 kcal/mol, indicating favorable interactions between quercetin and these proteins (Table 1; Fig 3A).

**Table 1:** Docking results of quercetin and 12 core target proteins.

proteintarget	docking			Resultkcal·mol-1
	*X*	*Y*	*Z*	
GSK3β (1Q5K)	23.14	22.18	9.398	−8.196
MAP2K1 (5BX0)	15.03	−26.189	−9.385	−8.515
SRC (7OTE)	5.585	−5.35	2.088	−9.922
KDR (3WZD)	4.818	3.013	19.51	−8.213
MAPK1 (4iz5)	−20.72	40.109	18.595	−8.388
AKT1 (3O96)	9.657	−7.76	10.604	−9.602
IGF1 (3O23)	7.479	1.242	21.691	−7.328
JAK2 (3KRR)	15.21	11.24	4.17	−9.586
HRAS (6q21)	5.266	35.062	91.95	−8.31
MET (1MJL)	−9.253	7.89	31.04	−6.389
EGFR (1M17)	21.698	0.303	52.092	−7.704
IGF1R (2OJ9)	5.896	−7.753	21.087	−7.843

To further validate these predictions, we examined the effect of quercetin on EGFR expression and downstream signaling by western blot analysis. After 24 h of quercetin treatment, HCC cells showed a significant reduction not only in total EGFR expression, but also in the phosphorylation level of EGFR (Fig 3B, C). Moreover, quercetin substantially reduced phosphorylation levels of key molecules in the PI3K/AKT/GSK3β axis, a pathway critically involved in cell cycle regulation and proliferation. These results suggest that quercetin may exert its anti-proliferative effects, at least in part, by targeting EGFR and suppressing its downstream PI3K/AKT/GSK3β signaling.

### 3.3. HBx promotes HCC proliferation and metastasis via EGFR activation

Accumulating evidence has established that chronic HBV infection is a principal etiological driver of HCC [36,37], with the HBx protein playing a central role in HBV-HCC [38]. To investigate the oncogenic function of HBx in HCC, we established stable HepG2 cell lines overexpressing HBx via lentiviral transduction. CCK-8 assays revealed a marked increase in the proliferative capacity of HBx-overexpressing cells (S1A Fig). This pro-proliferative effect was further corroborated by higher EdU-positive rates and enhanced colony formation efficiency in HepG2-HBx cells (S1B,C Fig). Collectively, these findings identify HBx as a significant promoter of HCC cell proliferation.

Scratch assays demonstrated accelerated closure in HepG2-HBx cells compared to the controls (S1D Fig). Consistently, transwell migration assays revealed enhanced migratory potential in HBx-overexpressing HCC cells (S1E Fig). These results suggest that HBx upregulation may potentiate the migratory capacity of HCC cells.

Mechanistically, HBx overexpression significantly upregulated both total EGFR and its phosphorylation, leading to activation of the downstream PI3K/AKT/GSK3β cascade, as reflected by increased phosphorylated PI3K, AKT, and GSK3β (S1F Fig). Furthermore, HBx-overexpressing cells exhibited reduced E-cadherin expression alongside elevated levels of N-cadherin and Vimentin, indicating induction of EMT (S1G Fig). Taken together, these findings suggest that HBx promotes HCC cell proliferation and migration, at least in part, by activating the EGFR/PI3K/AKT/GSK3β axis and subsequently inducing EMT.

### 3.4. HBV-HCC proliferation and migration are suppressed by quercetin

**Fig 4:**
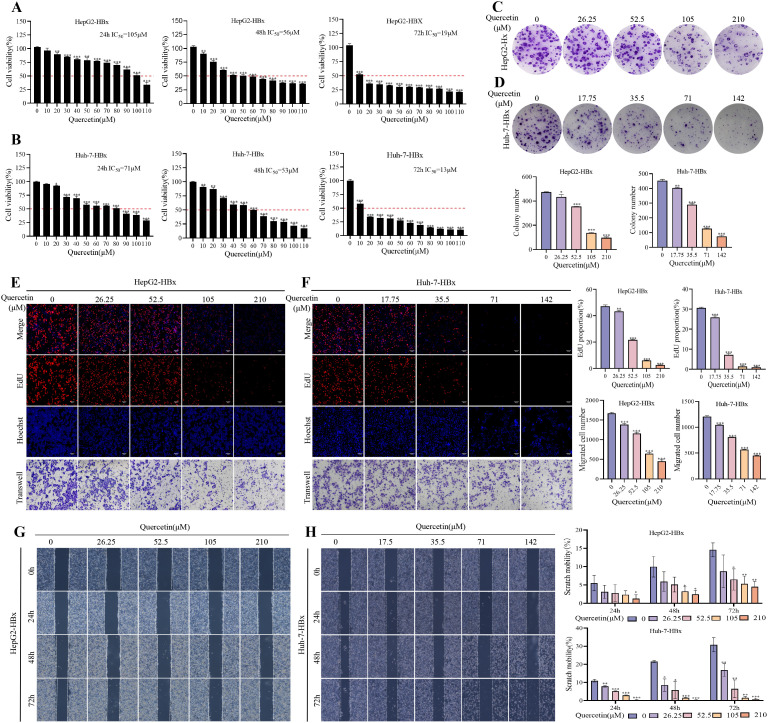
Quercetin inhibits the proliferation and migration of HBV-HCC cells in vitro. **(A-B)** The effect of quercetin at different concentrations on cell viability in HepG2-HBx and Huh-7-HBx cells after 24, 48, and 72 h of treatment was assessed by the CCK-8 assay. **(C-D)** Colony formation ability was evaluated using a colony formation assay after quercetin treatment at the indicated concentrations for 24 h. **(E-F)** Cell proliferation and migration were assessed using the EdU incorporation assay and Transwell assay, respectively, after quercetin treatment at the indicated concentrations for 24 h. **(G-H)** The migratory capacity of HBV-HCC cells was evaluated by a scratch assay at 0, 24, 48, and 72 h after quercetin treatment at the indicated concentrations. All experiments were performed in triplicate (n = 3). Data are presented as mean ± SD. Note: ^*^P < 0.05, ^**^P < 0.01, ^***^P < 0.001 vs. control group.

Building upon previous reports of quercetin’s anti-HCC activity, we specifically evaluated its efficacy against HBV-HCC using established HepG2-HBx and Huh-7-HBx cell models. Our results demonstrate that quercetin treatment produced concentration- and time-dependent inhibition of cell viability in both HBV-HCC cell lines, as measured by CCK-8 assays (Fig 4A, B). Based on the IC_50_ values of the two cell lines at 24 h, corresponding concentration gradients were established for subsequent experimental validation. Clonogenic assays indicated a dose-dependent reduction in colony formation capacity (Fig 4C, D). Furthermore, EdU incorporation analysis showed that quercetin dose-dependently restrained the proliferation of HBV-HCC, as evidenced by a reduced proportion of EdU-positive cells. Transwell migration assays demonstrated significant reductions in the number of migrated cells following treatment (Fig 4E, F). Consistently, scratch assays exhibited delayed wound closure speed in both cell models (Fig 4G, H). Collectively, these findings suggest that quercetin exhibits anti-migratory and anti-proliferative effects in HBV-HCC.

### 3.5. Quercetin inhibits HBV-HCC progression via EGFR-mediated suppression of PI3K/AKT/GSK3β signaling

**Fig 5:**
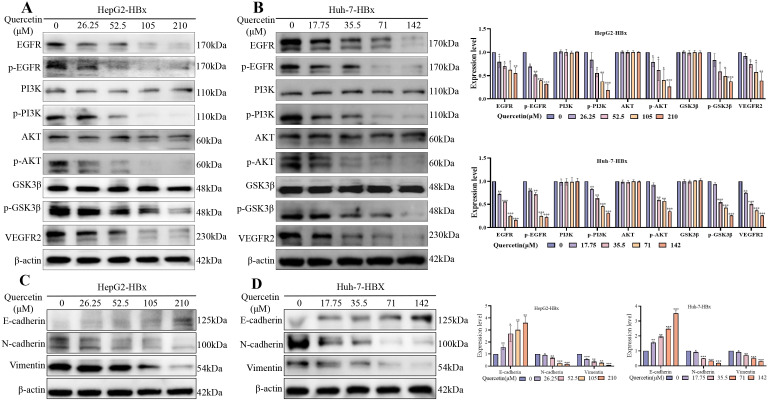
The inhibitory effect of quercetin on the expression of proteins related to HBV-HCC. **(A-B)** Western blot analysis demonstrated the effect of quercetin on the expression of proteins in the phosphorylated EGFR/PI3K/AKT/GSK3β pathway. **(C-D)** Effect of quercetin treatment on the expression of EMT-related proteins. All experiments were performed in triplicate (n = 3) after treatment for 24 h. Data are presented as mean ± SD. Note: ^*^P < 0.05, ^**^P < 0.01, ^***^P < 0.001 vs. control group.

Based on these findings, we observed that quercetin inhibited the growth of HBV-HCC cells. Western blot analysis demonstrated that quercetin treatment significantly reduced both total and phosphorylated EGFR levels in HBV-HCC cells, which was accompanied by markedly decreased phosphorylation of the downstream cell proliferation regulators PI3K, AKT, and GSK3β (Fig 5A, B). Moreover, quercetin treatment notably up-regulated the expression of the epithelial marker E-cadherin, while down-regulating the expression of the mesenchymal markers N-cadherin and Vimentin (Fig 5C, D), suggesting a suppression of the EMT process. Taken together, these results imply that EGFR may play a critical role in the inhibitory effects of quercetin on HBV-HCC progression.

**Fig 6:**
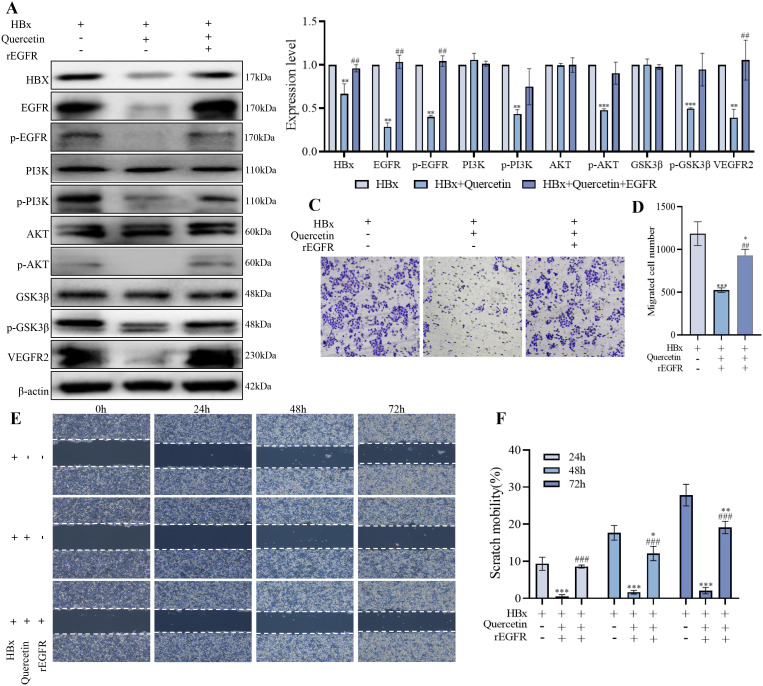
Quercetin inhibits HBV-HCC by targeting the EGFR. **(A-B)** Western blotting analysis was performed to evaluate the effect of EGFR overexpression on quercetin-induced downregulation of phosphorylated PI3K, AKT, and GSK3β after treatment at the indicated concentrations for 24 h. **(C-D)** Cell migration was assessed using a Transwell assay after EGFR overexpression and quercetin treatment at the indicated concentrations for 24 h. **(E-F)** The migratory capacity of HBV-HCC cells was evaluated by a scratch assay at 0, 24, 48, and 72 h after EGFR overexpression and quercetin treatment. All experiments were performed in triplicate (n = 3). Data are presented as mean ± SD. Note: ^*^P < 0.05, ^**^P < 0.01, ^***^P < 0.001 vs. the HBx group; and ^#^*P* < 0.05, ^##^*P* < 0.01, ^###^*P* < 0.001 vs. the Quercetin group.

To investigate the mechanistic role of EGFR in quercetin’s anti-tumor effects, we established EGFR-overexpressing HepG2-HBx cell lines. Western blot analysis showed that EGFR overexpression not only restored quercetin-suppressed EGFR phosphorylation, but also reversed the downstream inhibition of the PI3K/AKT/GSK3β axis, as reflected by the recovered phosphorylation levels of PI3K, AKT, and GSK3β (Fig 6A, B).

Transwell migration assays further demonstrated that EGFR overexpression substantially enhanced cellular migratory capacity compared to the quercetin-treated group (Fig 6C, D). Similarly, scratch assays indicated superior migration and wound closure capabilities in EGFR-overexpressing HBV-HCC cells compared to quercetin-treated controls (Fig 6E, F). These results provide compelling evidence that quercetin exerts its anti-proliferative and anti-migratory effects in HBV-HCC primarily through EGFR targeting, leading to subsequent inhibition of the PI3K/AKT/GSK3β axis. The complete reversal of quercetin’s therapeutic effects upon EGFR overexpression strongly supports EGFR as the critical molecular target mediating quercetin’s anti-tumor activity in this context.

### 3.6. Quercetin suppresses tumor growth in HBV-HCC xenograft models through EGFR pathway inhibition

**Fig 7:**
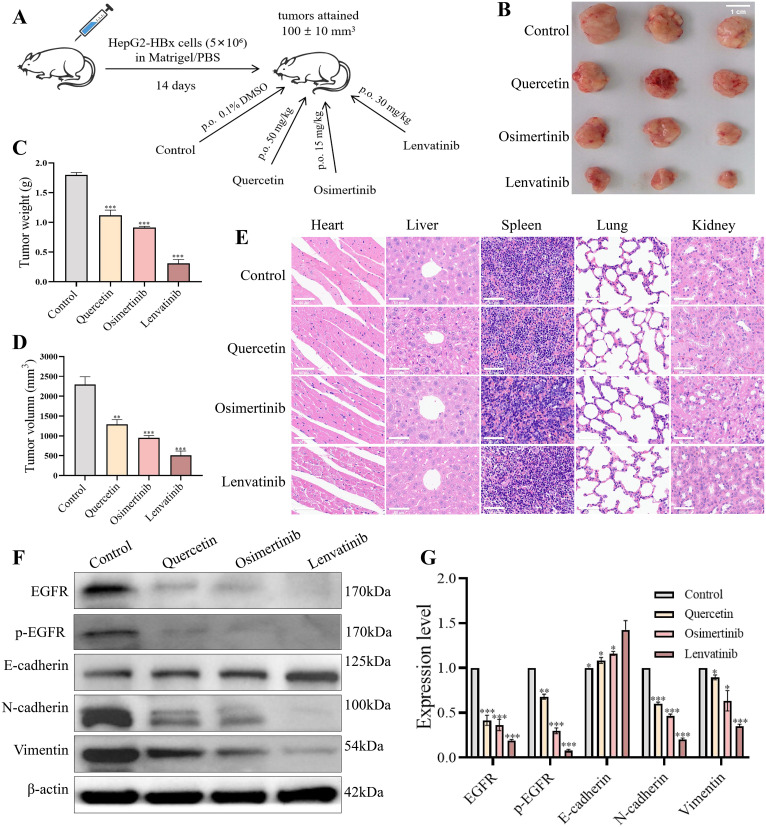
Quercetin suppresses tumor growth in vivo. **(A)** Schematic diagram of experimental mouse model construction and drug intervention plan. **(B)** Representative photographs of excised tumor specimens collected post-euthanasia. **(C)** Statistical comparison of tumor mass (g) among different drug treatment groups. **(D)** Statistical comparison of tumor volume (mm³) across drug treatment groups. **(E)** H&E staining of heart, liver, spleen, lung, and kidney tissues from healthy mice treated with quercetin. **(F-G)** The expression levels of EGFR, E-cadherin, N-cadherin and Vimentin in tumor tissues of tumor-bearing mice treated with quercetin, osimertinib or lenvatinib were analyzed by Western Blot. Note: ^*^P < 0.05, ^**^P < 0.01, ^***^P < 0.001 (vs. respective control groups).

Building on in vitro anti-tumor activity of quercetin against HBV-HCC cells, we evaluated its therapeutic efficacy in BALB/c nude mouse xenograft models (Fig 7A). Both quercetin and the positive control drug lenvatinib treatment significantly reduced tumor volume compared to the control group (Fig 7B-D), indicating that quercetin suppresses tumor growth in vivo. To further explore the EGFR pathway, we employed osimertinib, a third-generation EGFR tyrosine kinase inhibitor, which similarly demonstrated potent tumor growth suppression. Furthermore, H&E staining of major organs revealed no evidence of drug-induced toxicity (Fig 7E). To validate the mechanism of action in vivo, we examined the expression levels of EGFR, E-cadherin, N-cadherin, and Vimentin in tumor tissues by Western blot (Fig 7F, G). Compared with the control group, both quercetin and the EGFR inhibitor osimertinib significantly reduced EGFR protein expression, downregulated N-cadherin and Vimentin, and upregulated E-cadherin. These findings indicate that quercetin inhibits HBV-HCC growth in vivo, at least in part, by suppressing the EGFR signaling pathway and reversing the EMT process.

**Fig 8:**
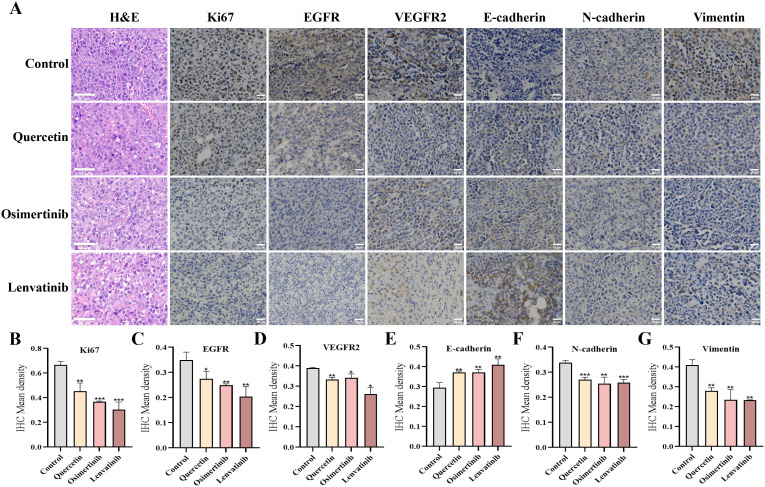
H&E and IHC staining of tumor tissues after treatment with quercetin, osimertinib, or lenvatinib. **(A)** H&E staining and immunohistochemical analysis of Ki67, EGFR, VEGFR2, E-cadherin, N-cadherin, and Vimentin expression in tumor tissues from tumor-bearing mice following intervention with quercetin, osimertinib, or lenvatinib. **(B-G)** Statistical analysis of immunohistochemical staining of mouse tumor tissues. Note: ^*^P < 0.05, ^**^P < 0.01, ^***^P < 0.001 (vs. respective control groups).

H&E-stained tumor sections revealed that the neoplasms in the control group exhibited hyperchromatic nuclei, cellular hypertrophy, increased proliferative activity, and a densely packed architectural arrangement. In contrast, tumors treated with quercetin, osimertinib, or lenvatinib exhibited reduced volume, vacuolated architecture, and prominent nuclear pyknosis, histological features collectively indicative of suppressed tumor cell proliferation. Immunohistochemical analysis further demonstrated a significant decrease in the proportion of Ki67-positive cells following treatment with quercetin, osimertinib, or lenvatinib (Fig 8A-G). Consistent with the Western Blot detection results, EGFR expression was downregulated in all treatment groups compared to the control. Additionally, quercetin treatment significantly reversed the EMT process, as evidenced by upregulated E-cadherin and downregulated N-cadherin and Vimentin expression. Collectively, these results substantiate that quercetin curbs HBV-HCC progression in vivo by targeting the EGFR and reversing EMT.

## 4. Discussion

This study provides several novel insights into the molecular mechanisms underlying quercetin’s anti-HBV-HCC activity by integrating network pharmacology, molecular docking, and rigorous experimental validation. First, we established a mechanistic framework linking the HBV oncoprotein HBx to aberrant activation of the EGFR/PI3K/AKT/GSK3β signaling axis in HCC cells. Within this disease‑relevant context, we demonstrated that quercetin effectively counteracts HBx-driven EGFR activation and downstream oncogenic signaling, a finding that extends beyond its generally reported anti-HCC effects. Furthermore, our EGFR overexpression rescue experiments provided direct functional evidence that EGFR is not merely a correlative biomarker, but a critical therapeutic target through which quercetin exerts its tumor‑suppressive effects in HBV-associated HCC. These findings collectively delineate a specific, target‑driven mechanism, offering a new perspective on quercetin’s therapeutic potential that is distinct from its broad multi‑target activity previously described.

HBx, a transactivator protein encoded by HBV, plays a pivotal role in HBV-HCC pathogenesis by driving viral replication and exerting direct oncogenic effects [39]. Its oncogenic mechanisms involve SIRT2 signaling pathway [40] and aberrant gene expression that directly promotes HCC [41]. Consistent with these findings, our study demonstrated that HBx overexpression significantly enhanced the propagation and migration of HCC cells. Importantly, HBx has been documented to upregulate EGFR expression [42], a receptor tyrosine kinase frequently overexpressed in HCC and associated with clinical prognoses [43]. Mechanistically, EGFR drives HCC progression through multiple oncogenic pathways, including the RAS/RAF/MEK/ERK cascade, STAT3 signaling, and the PI3K/AKT axis, which collectively promote cell cycle progression, inhibit apoptosis, facilitate immune evasion, and enhance tumor cell motility [44–47]. Interestingly, previous studies have reported context-dependent regulatory effects of HBx on EGFR expression, including both upregulation and downregulation through distinct molecular mechanisms, suggesting a complex and dynamic regulatory relationship. Our results elucidate that HBx-induced EGFR activation triggers the PI3K/AKT/GSK3β signaling cascade. Mechanistically, EGFR recruits PI3K to convert PIP2 to PIP3, leading to AKT phosphorylation and subsequent inactivation of GSK3β via Ser9 phosphorylation [48,49]. This axis ultimately promotes oncogenic processes including cell survival, invasion, metastasis and EMT. Collectively, these findings provide mechanistic evidence supporting EGFR as a promising therapeutic target in HBV-HCC and lay the groundwork for investigating targeted interventions, such as the natural compound quercetin, which we explore in this study.

Quercetin, a naturally occurring flavonoid widely distributed in foods, exerts multiple beneficial effects, including antioxidant, anti-inflammatory, and cytoprotective activities [50–52]. Recent studies have demonstrated its therapeutic potential against HCC [53,54], prompting further investigation into its mechanism of action in HBV-HCC. Our preliminary experiments demonstrated that quercetin dose-dependently suppresses the proliferation and migration of HBV-HCC cells,accompanied by upregulation of E-cadherin and downregulation of N-cadherin and Vimentin, suggesting its potential to reverse EMT. To elucidate the underlying mechanism, we integrated network pharmacology prediction, molecular docking, and experimental validation. Network pharmacology prediction identified EGFR as a key molecular target, and molecular docking revealed a strong binding affinity between quercetin and EGFR (binding free energy = −7.704 kcal·mol ⁻ ¹). These in silico predictions were corroborated by Western blot analysis, which showed that quercetin downregulated EGFR expression and suppressed the downstream PI3K/AKT/GSK3β axis in HBV-HCC cells. Importantly, this inhibitory effect was significantly reversed by EGFR overexpression, confirming the functional dependence of quercetin’s anti-tumor activity on EGFR targeting. These findings align with accumulating evidence that the PI3K/AKT/GSK3β axis promotes HCC progression through the regulation of cell cycle-related proteins [55]. The therapeutic relevance of this mechanism was further supported by in vivo immunohistochemical analysis, which demonstrated that quercetin significantly reduced EGFR expression in tumor tissues, with efficacy comparable to conventional EGFR inhibitors. It is well recognized that quercetin, like many flavonoids, exhibits low oral bioavailability due to poor water solubility and extensive first-pass metabolism [56]. Nevertheless, orally administered quercetin has consistently demonstrated significant in vivo anti-tumor efficacy across numerous studies—a phenomenon often referred to as the “low bioavailability/high bioactivity” paradox. Notably, recent evidence suggests that gut microbiota-derived metabolites of quercetin, such as DOPAC, can exert potent anti-tumor effects via immunomodulatory mechanisms [57], providing a plausible explanation for the efficacy observed in our xenograft model despite the low systemic exposure of the parent compound. Notably, while conventional EGFR inhibitors are often associated with drug resistance, limited efficacy, adverse effects, and high costs, quercetin—as a multi-target natural compound—offers potential advantages, including a favorable safety profile and cost-effectiveness. In summary, this study provides mechanistic evidence that quercetin inhibits HBV-HCC progression through EGFR-mediated suppression of the PI3K/AKT/GSK3β axis and modulation of EMT. These findings position quercetin as a promising candidate for further development as a targeted therapeutic agent in HBV-HCC.

Importantly, it should be noted that quercetin is widely recognized as a multi-target compound. Consistent with our network pharmacology and molecular docking results, multiple potential targets—including SRC, AKT1, MAPK1, and IGF1R—were identified in addition to EGFR. Although EGFR was selected for experimental validation due to its central role in the protein–protein interaction network and its upstream regulatory position in key oncogenic signaling pathways, we do not exclude the possibility that other targets also contribute to the observed anti-tumor effects of quercetin. Notably, EGFR overexpression significantly attenuated the inhibitory effects of quercetin in our functional assays, supporting its role as a key mediator; however, this does not fully exclude the involvement of parallel signaling pathways or additional molecular targets. Therefore, quercetin’s anti-HBV-HCC activity is likely mediated through a coordinated multi-target regulatory mechanism rather than a single-target effect. Future studies integrating multi-target validation strategies will be essential to further delineate the complex pharmacological network underlying quercetin’s therapeutic effects. While the present study provides mechanistic evidence supporting EGFR as a key mediator of quercetin’s anti-tumor effects in HBV-HCC, several important aspects of EGFR signaling regulation remain to be further elucidated. In this study, we primarily focused on total EGFR expression and its downstream PI3K/AKT/GSK3β axis, which was consistently supported by molecular docking predictions, Western blot validation, and functional rescue experiments. However, EGFR activation is a highly dynamic process that is tightly regulated by phosphorylation at specific tyrosine residues. The absence of direct assessment of EGFR phosphorylation represents a limitation of the current study, and future investigations incorporating phosphorylation-specific analyses will be essential to fully clarify the impact of quercetin on EGFR activation status. In addition to the PI3K/AKT pathway, EGFR is known to activate multiple downstream signaling cascades, among which the RAS/RAF/MEK/ERK pathway plays a critical role in tumor proliferation and survival. Although our results clearly demonstrate that quercetin suppresses EGFR expression and downstream PI3K/AKT/GSK3β signaling, the potential involvement of ERK signaling was not evaluated in this study. Future studies should therefore include systematic analysis of ERK activation to provide a more comprehensive understanding of EGFR-mediated signaling networks regulated by quercetin. Furthermore, while quercetin-induced downregulation of EGFR protein levels was consistently observed, the underlying regulatory mechanism remains unclear. It is currently unknown whether this effect is mediated through transcriptional suppression, enhanced protein degradation, or modulation of protein stability. In particular, cycloheximide (CHX) chase assays and ubiquitination-related analyses would be valuable to determine whether quercetin affects EGFR protein turnover. Elucidating these mechanisms will be critical for understanding how quercetin regulates EGFR at the post-translational level.

Taken together, these limitations highlight important directions for future research and further refinement of the mechanistic framework. Despite these limitations, the present study provides robust evidence that quercetin exerts anti-tumor effects in HBV-HCC primarily through EGFR-dependent suppression of the PI3K/AKT/GSK3β signaling axis.

## 5. Conclusion

This study demonstrates that HBx drives HCC cell proliferation and migration via activation of the EGFR axis. Moreover, we elucidate the molecular basis underlying the anti-HBV-HCC activity of quercetin, a naturally occurring dietary flavonoid. Mechanistically, quercetin targets EGFR, leading to inhibition of the PI3K/AKT/GSK3β signaling pathway and reversal of EMT, thereby attenuating HBV-HCC progression. Collectively, these findings provide novel mechanistic insights and establish a theoretical foundation for the use of quercetin as a potential adjunctive therapy in HBV-HCC.

## Supporting information

S1 Fig HBx promotes proliferation and migration in HCC. (A) The proliferative capacity of HepG2 and HepG2-HBx cells was determined by the CCK-8 assay after culture for 24, 48, and 72 h. (B) Cell proliferation was assessed using the EdU incorporation assay after 24 h. (C) Colony formation ability was evaluated using a colony formation assay after 24 h. (D) The migratory ability of HCC cells was evaluated by a scratch assay at 0, 24, 48, and 72 h post-scratch. (E) Cell migration was assessed using a Transwell assay after 24 h. (F-G) Western blot analysis was performed to evaluate the expression of phosphorylated EGFR, PI3K, AKT, GSK3β, and EMT-related proteins (E-cadherin, N-cadherin, Vimentin) after 24 h. All experiments were performed in triplicate (n  =  3). Data are presented as mean  ±  SD. Note: *P  <  0.05, **P  <  0.01, ***P  <  0.001 vs. control group.
(TIF)

S1 Table A complete list of the 227 potential targets of quercetin and the 111 overlapping targets with HCC disease targets. (XLSX)

S2 Table The raw data, as well as the corresponding means and standard deviations. (XLSX)

S2 Fig Raw western blot data. (PDF)

S3 Fig Graphical Abstract. (PDF)

## Abbreviations

HCC: Hepatocellular Carcinoma
HBV: Hepatitis B Virus
HBx: Hepatitis B Virus X Protein
EGFR: Epidermal Growth Factor Receptor
VEGFR2: Vascular Endothelial Growth Factor Receptor 2
TCM: Traditional Chinese Medicine
GO: Gene Ontology
KEGG: Kyoto Encyclopedia of Genes and Genomes
DMSO: Dimethyl sulfoxide
H&E: Hematoxylin-eosin
IHC: Immunohistochemistry
PI3K: Phosphatidylinositol 3-Kinase
p-PI3K: Phosphorylated phosphatidylinositol-3-kinase
AKT: Protein Kinase B
p-AKT: Phosphorylated Protein Kinase B
GSK3β: glycogen synthase kinase-3β
p-GSK3β: Phosphorylated glycogen synthase kinase-3β
EMT: Epithelial-Mesenchymal Transition
PIP2: Phosphatidylinositol 4,5-bisphosphate
PIP3: Phosphatidylinositol-3,4,5-trisphosphate
VEGFR2: Vascular Endothelial Growth Factor Receptor 2
JAK2: Janus kinase 2
ERK: Extracellular Signal-Regulated Protein Kinases
MAPK: Mitogen-Activated Protein Kinase
STAT3: Signal Transducer And Activator Of Transcription 3
SIRT2: Sirtuin 2
mTOR: mammalian target of rapamycin.
