# Supplementary figures and images for: Quercetin suppresses the progression of HBV-associated hepatocellular carcinoma by modulating the EGFR signaling pathway
Source: PLoS One. 2026 Jun 12;21(6):e0350584. doi: 10.1371/journal.pone.0350584 (PMC13262952; doi:10.1371/journal.pone.0350584)

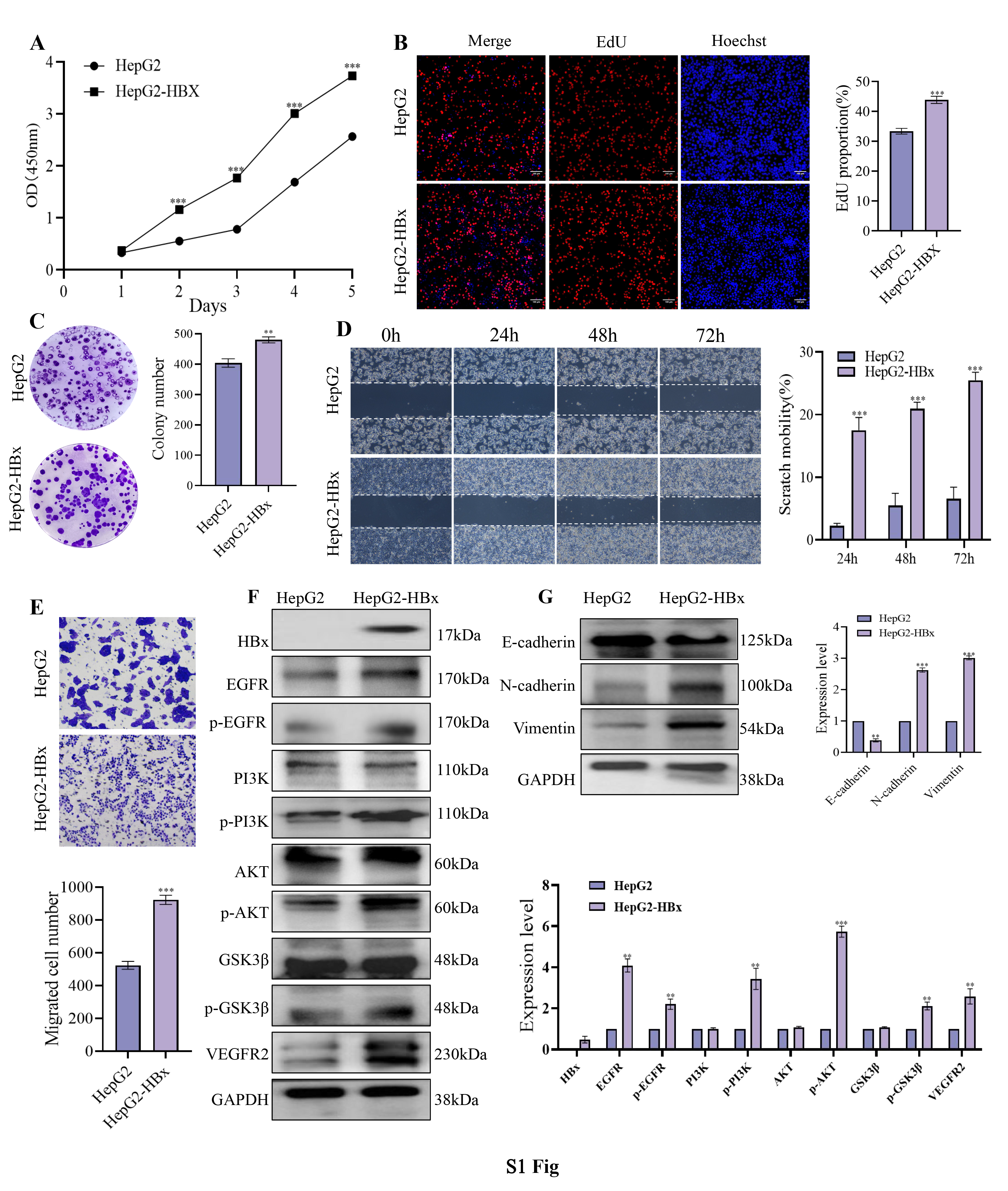

Supplement: S1 Fig — (A) The proliferative capacity of HepG2 and HepG2-HBx cells was determined by the CCK-8 assay after culture for 24, 48, and 72 h. (B) Cell proliferation was assessed using the EdU incorporation assay after 24 h. (C) Colony formation ability was evaluated using a colony formation assay after 24 h. (D) The migratory ability of HCC cells was evaluated by a scratch assay at 0, 24, 48, and 72 h post-scratch. (E) Cell migration was assessed using a Transwell assay after 24 h. (F-G) Western blot analysis was performed to evaluate the expression of phosphorylated EGFR, PI3K, AKT, GSK3β, and EMT-related proteins (E-cadherin, N-cadherin, Vimentin) after 24 h. All experiments were performed in triplicate (n = 3). Data are presented as mean ± SD. Note: *P < 0.05, **P < 0.01, ***P < 0.001 vs. control group. (TIF) [file pone.0350584.s001.tif]

Graphical Abstract

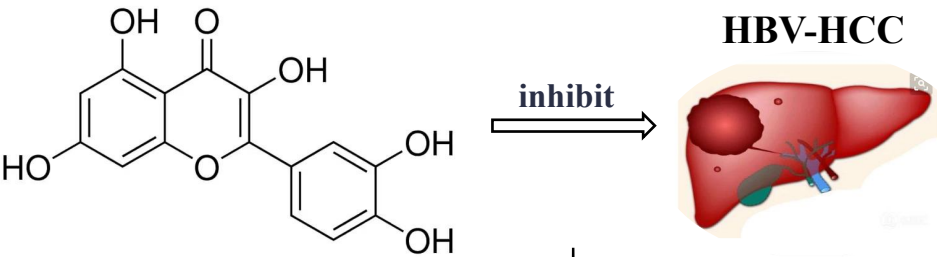

Network pharmacology analysis

In vitro

In vivo

Quercetin

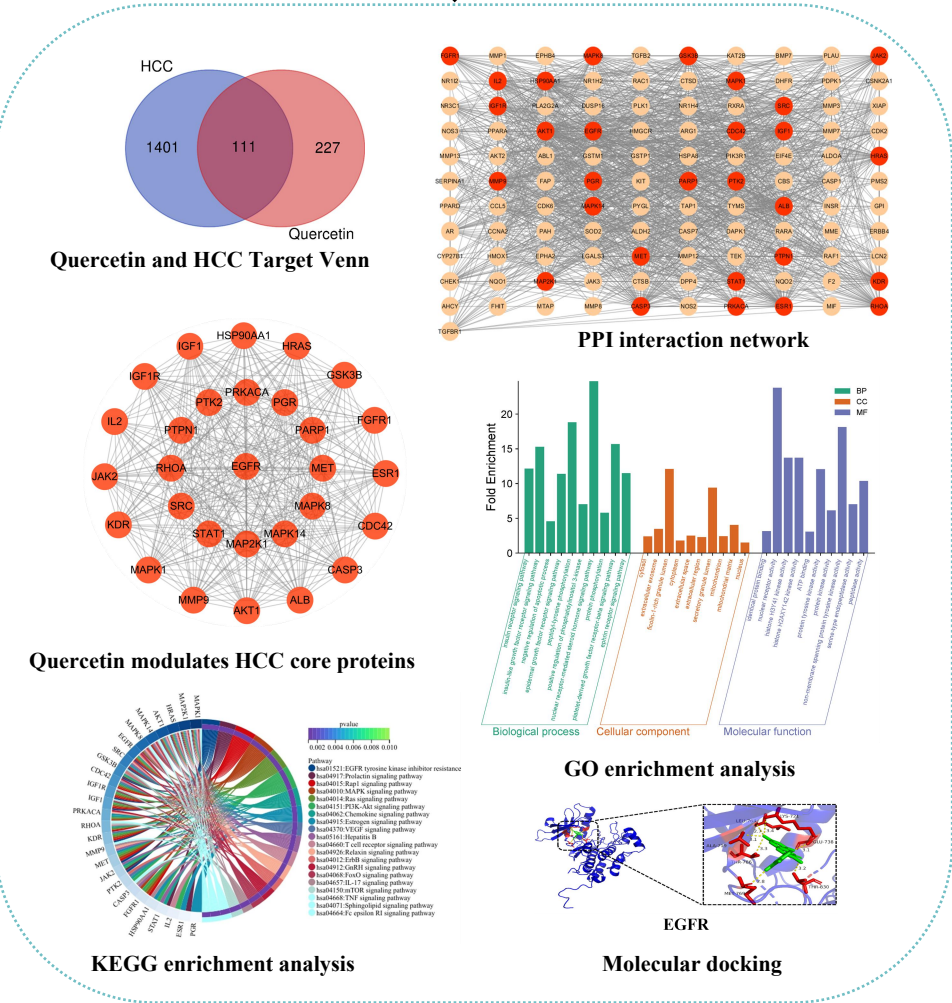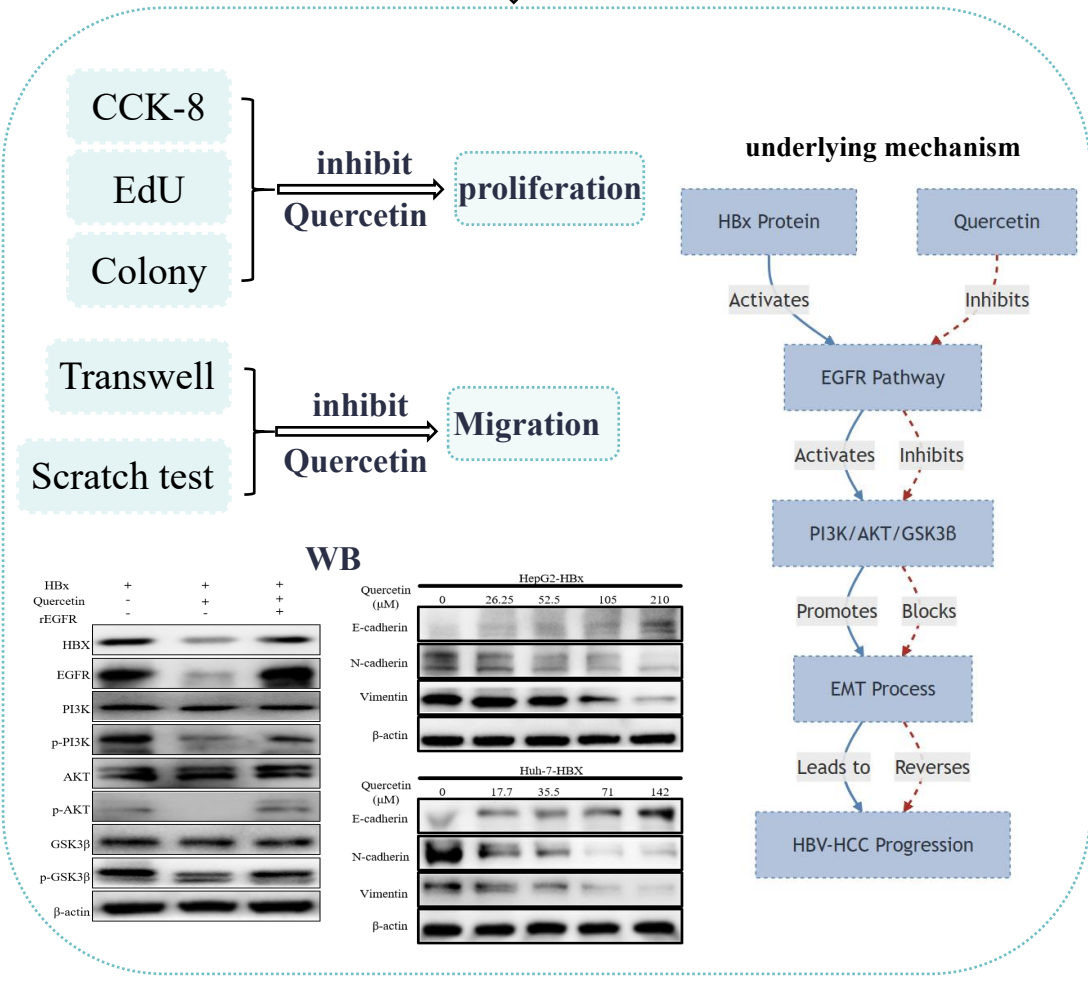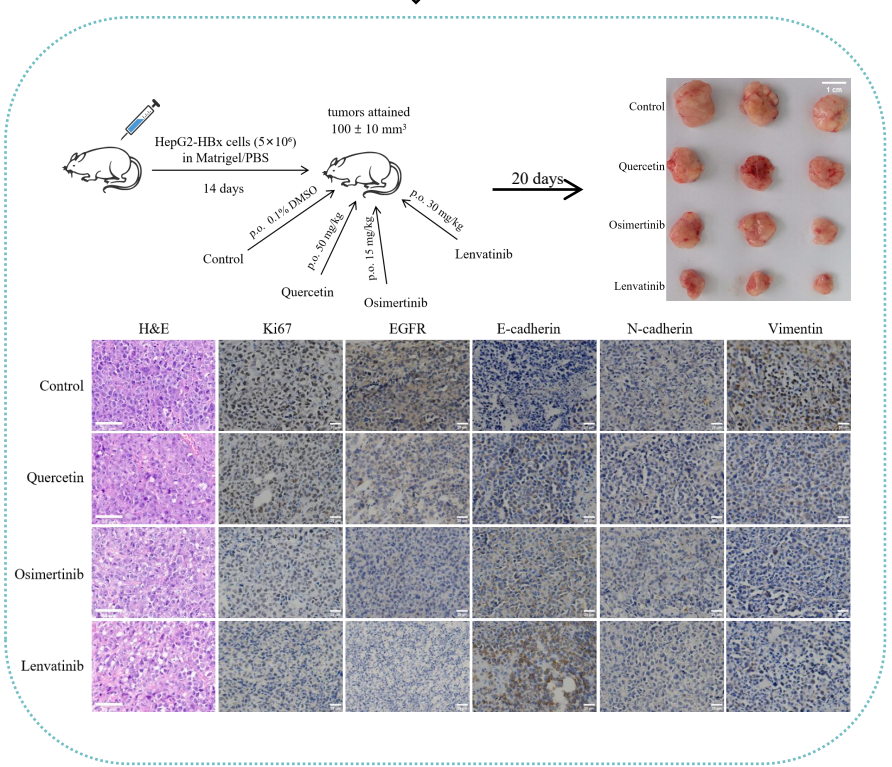

Supplement: S3 Fig — (PDF) [file pone.0350584.s005.pdf]
