# Supplementary material for: Quercetin suppresses the progression of HBV-associated hepatocellular carcinoma by modulating the EGFR signaling pathway
Source: PLoS One. 2026 Jun 12;21(6):e0350584. doi: 10.1371/journal.pone.0350584 (PMC13262952; doi:10.1371/journal.pone.0350584)

Figure 3B **HepG2**

Repeat 1

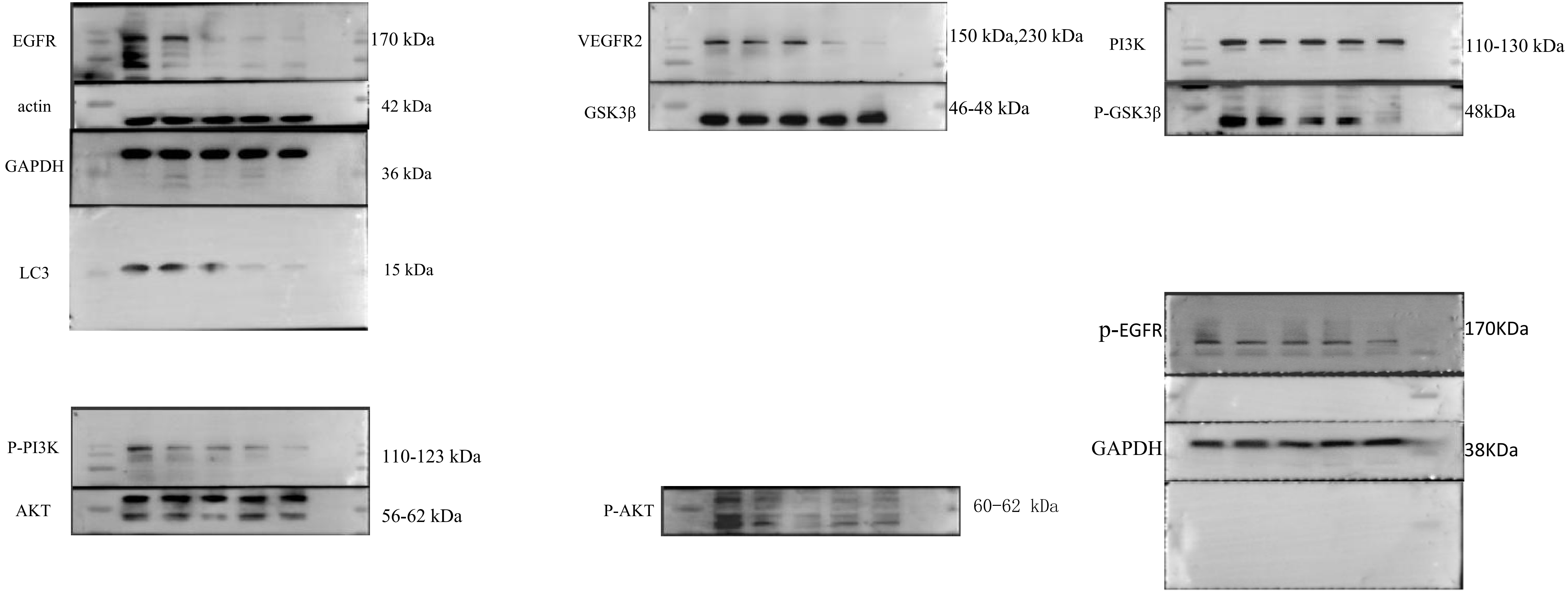

Figure 3B HepG2

Repeat 2

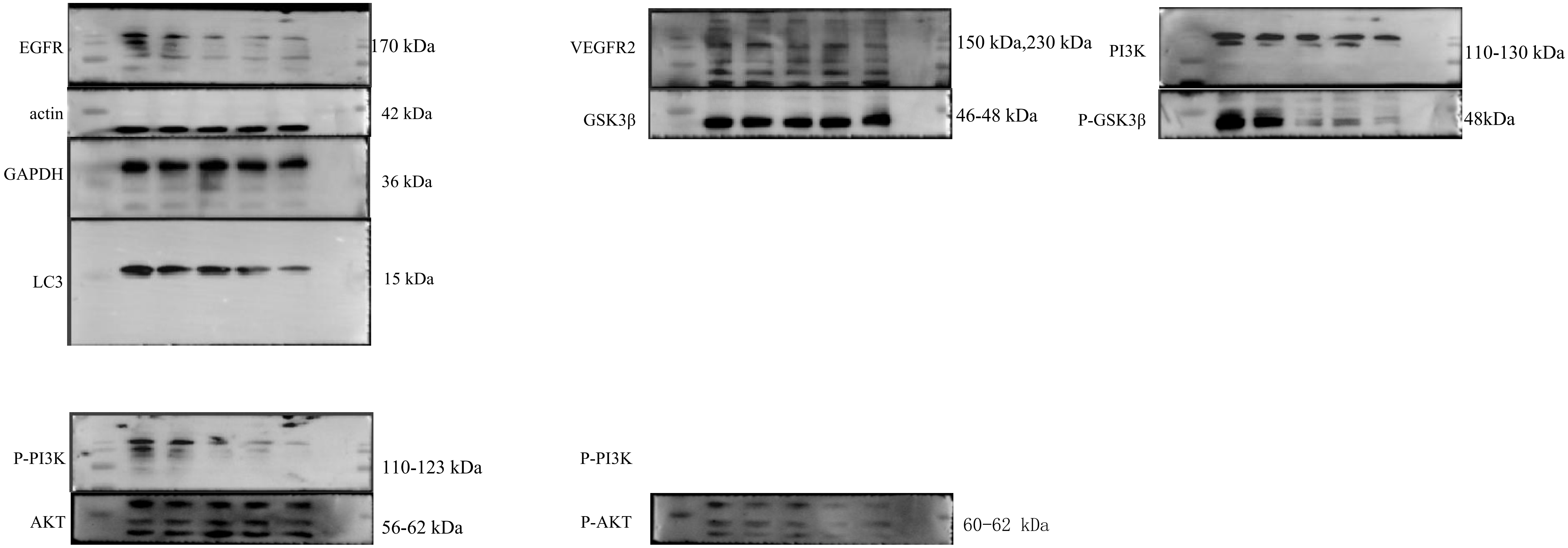

Figure 3B HepG2

Repeat3

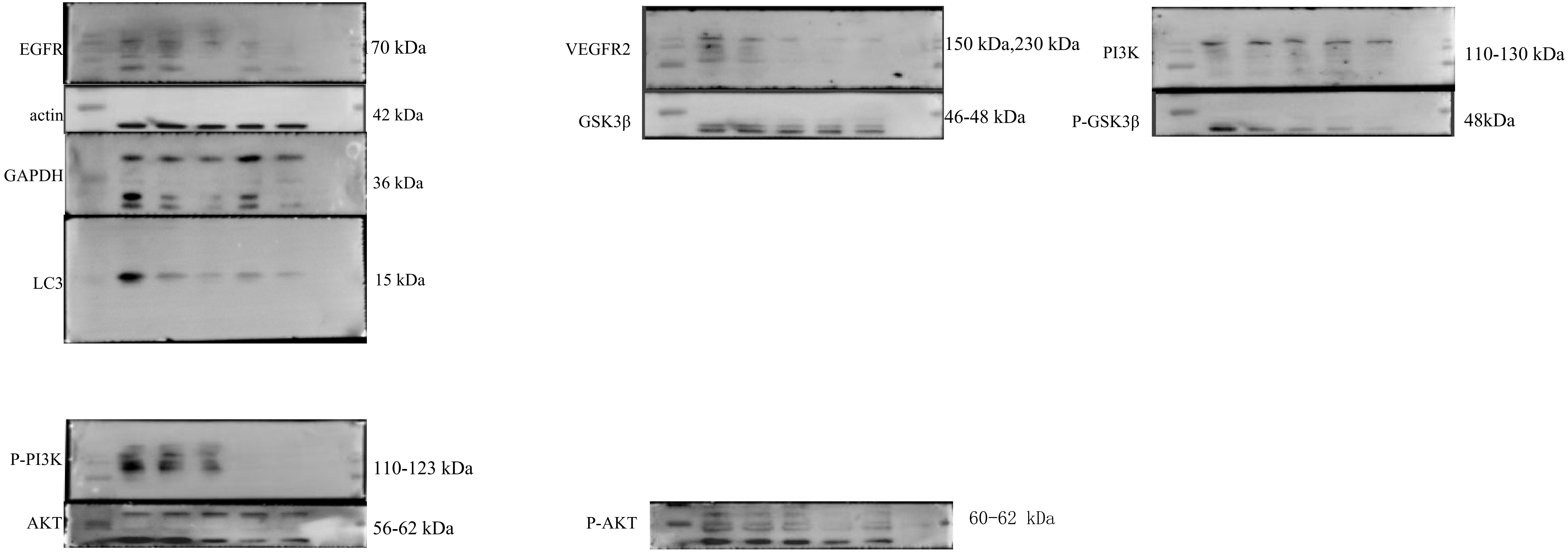

Figure 3C **Huh-7** **Repeat 1**

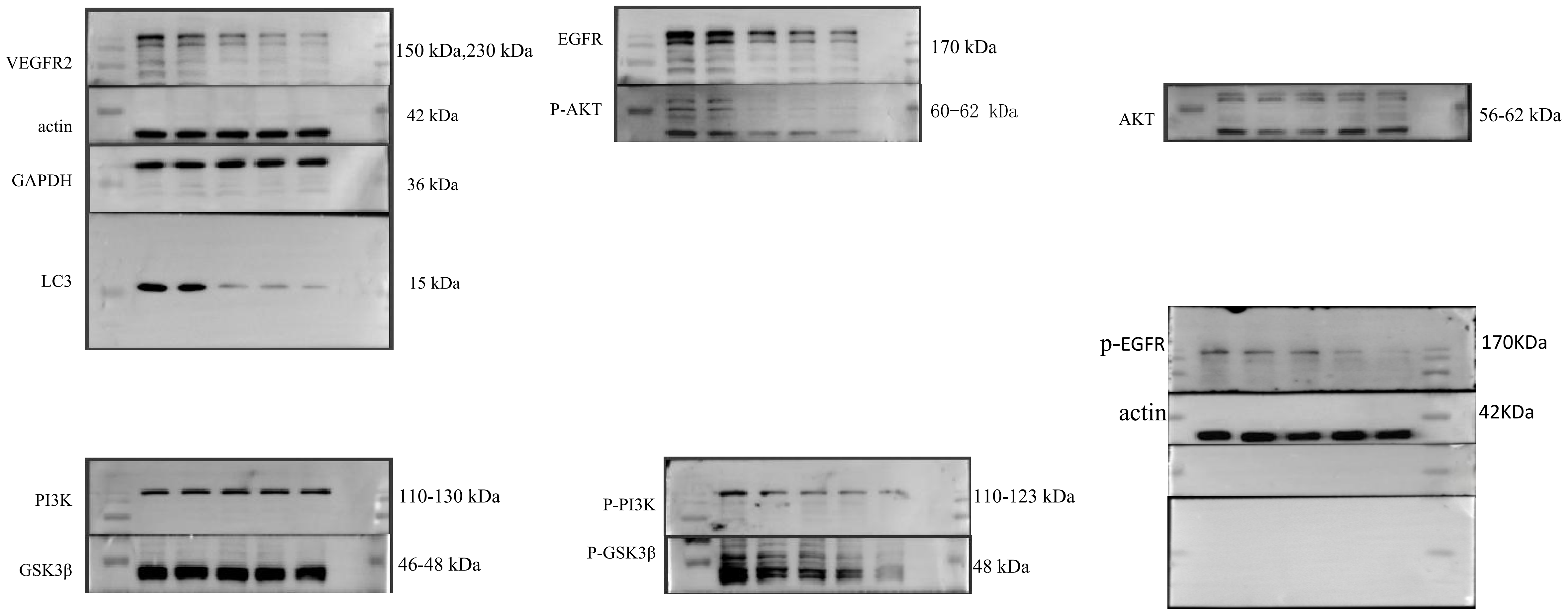

Repeat 2

Figure 3C **Huh-7**

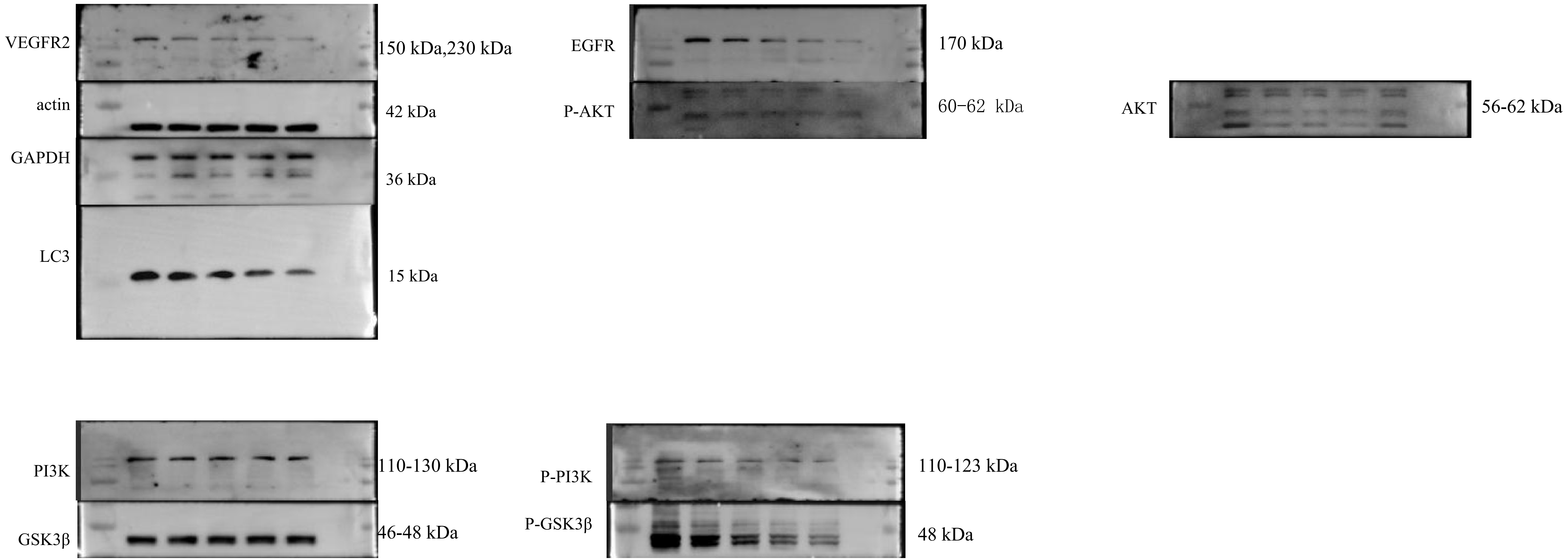

Figure 3C **Huh-7**

Repeat 3

VEGFR2
actin
GAPDH
LC3

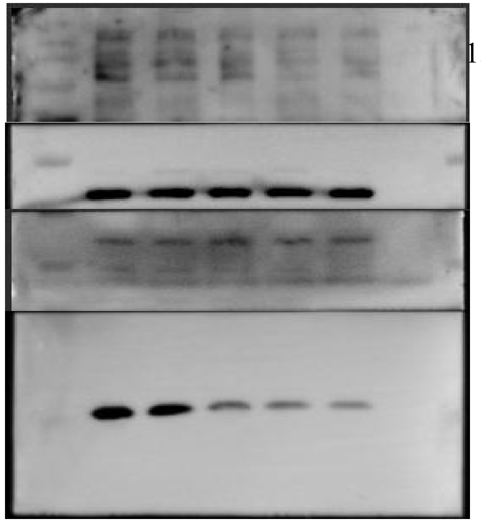

150 kDa,230 kDa
42 kDa
36 kDa
15 kDa

EGFR
P-AKT

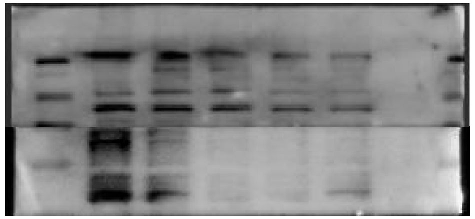

170 kDa
60-62 kDa

AKT

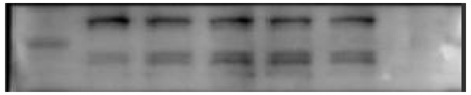

56-62 kDa

PI3K
GSK3β

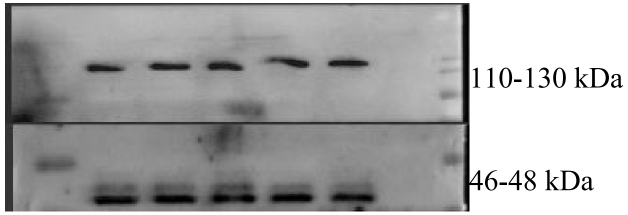

110-130 kDa
46-48 kDa

P-PI3K
P-GSK3β

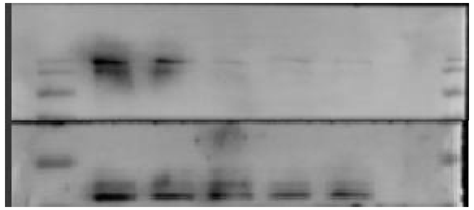

110-123 kDa
48 kDa

Figure 5A **HepG2-HBx**

Repeat 1

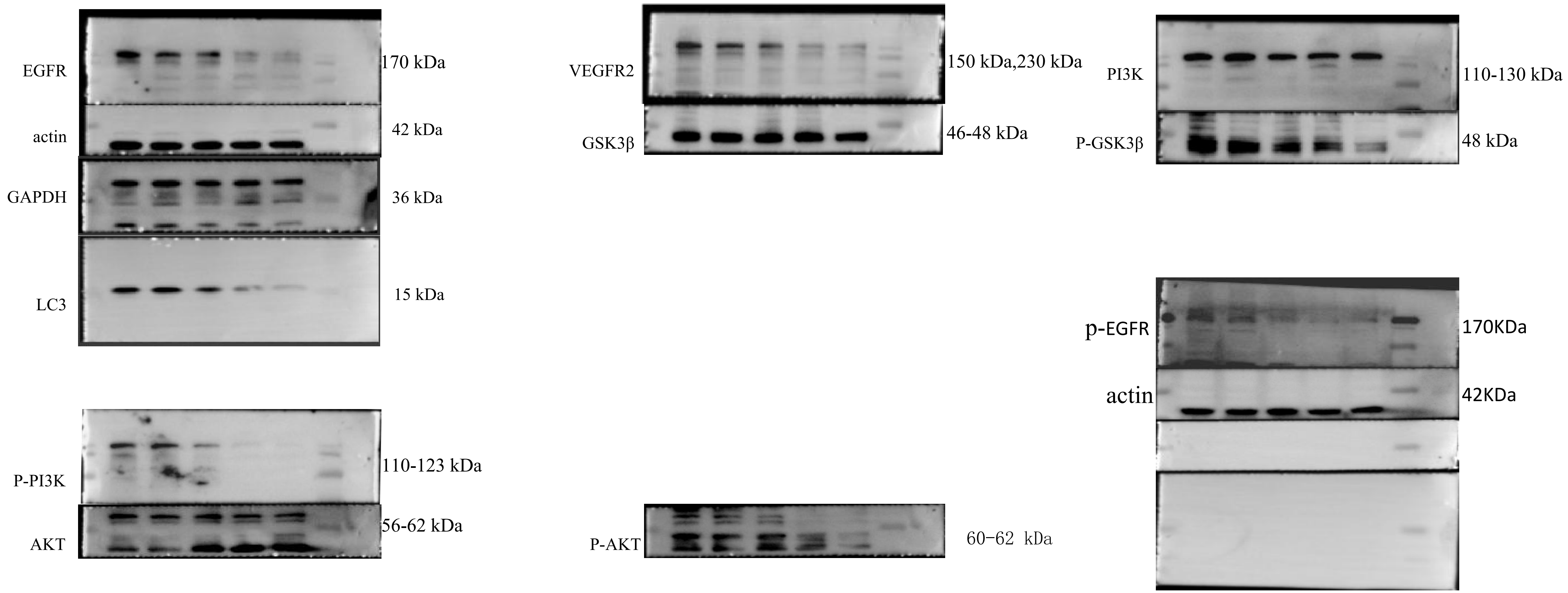

Figure 5A **HepG2-HBx**

Repeat 2

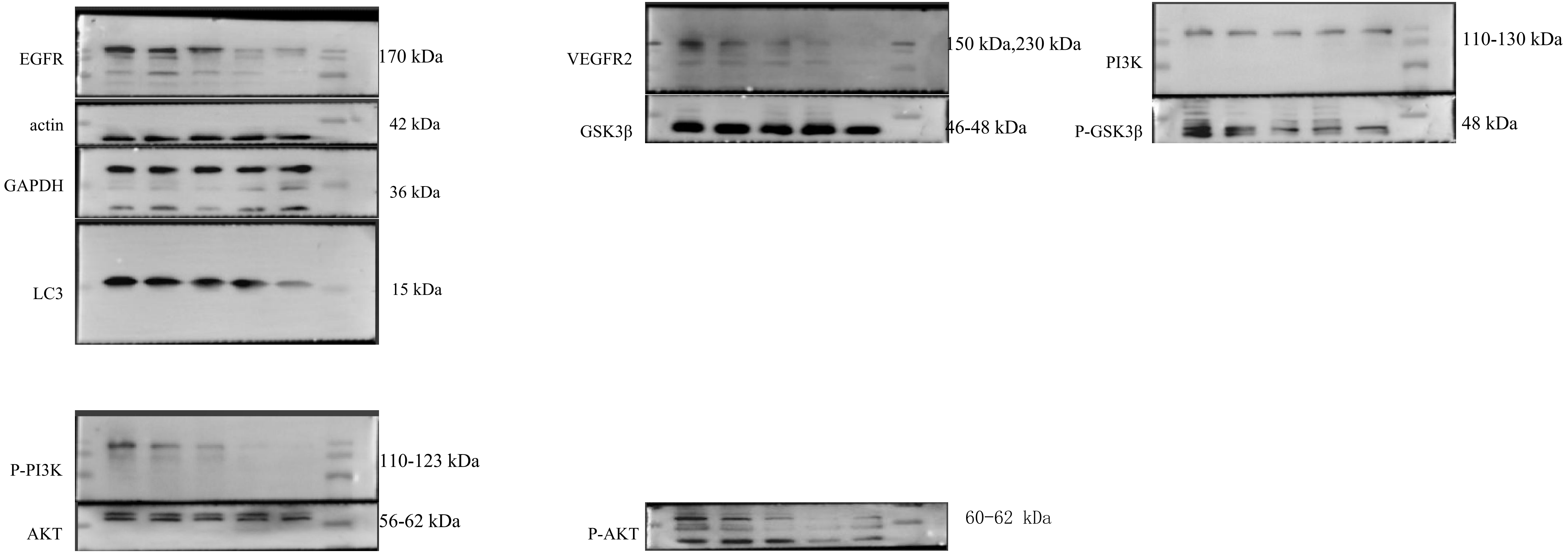

Figure 5A **HepG2-HBx** **Repeat 3**

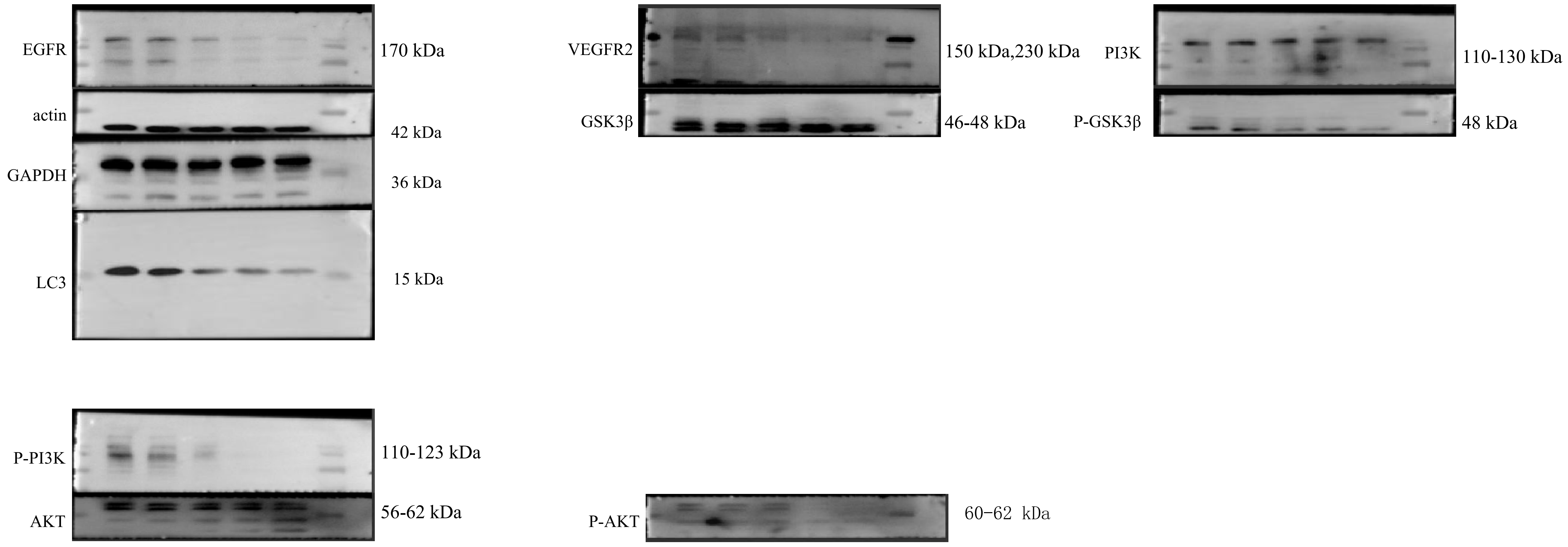

Figure 5B Huh-7-HBx

Repeat 1

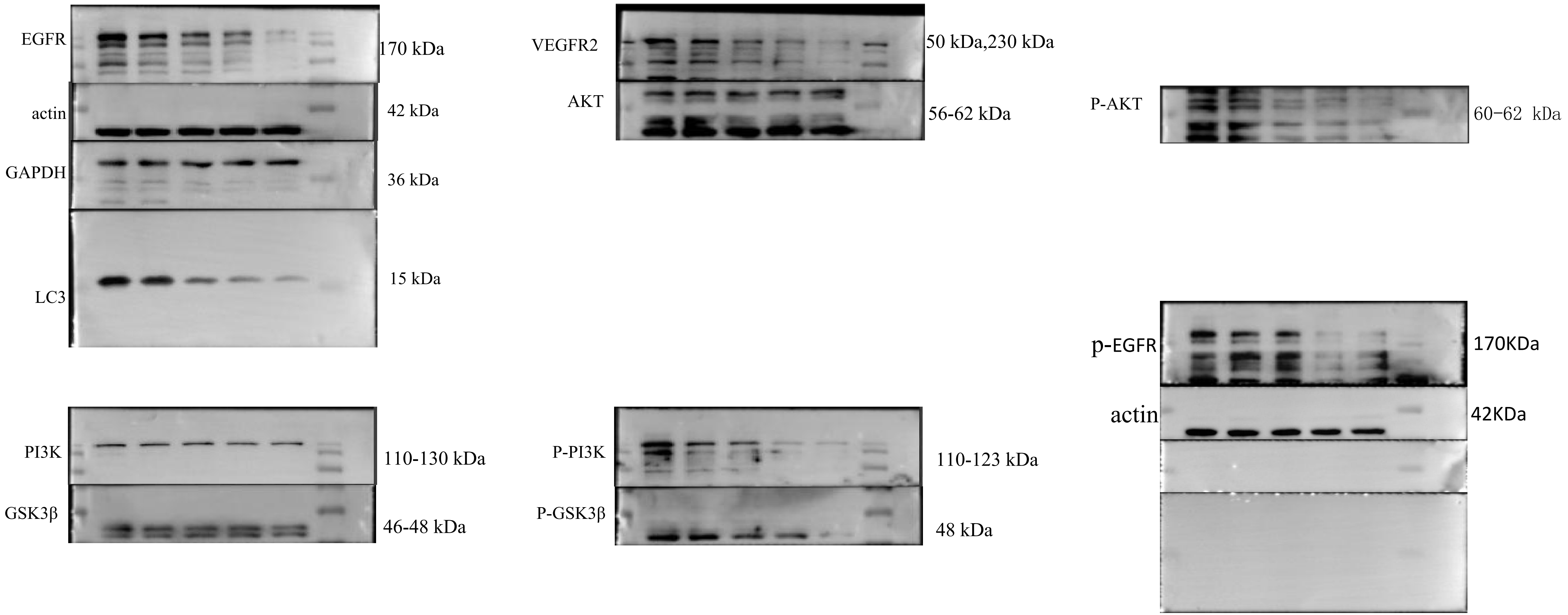

Figure 5B Huh-7-HBx

Repeat 2

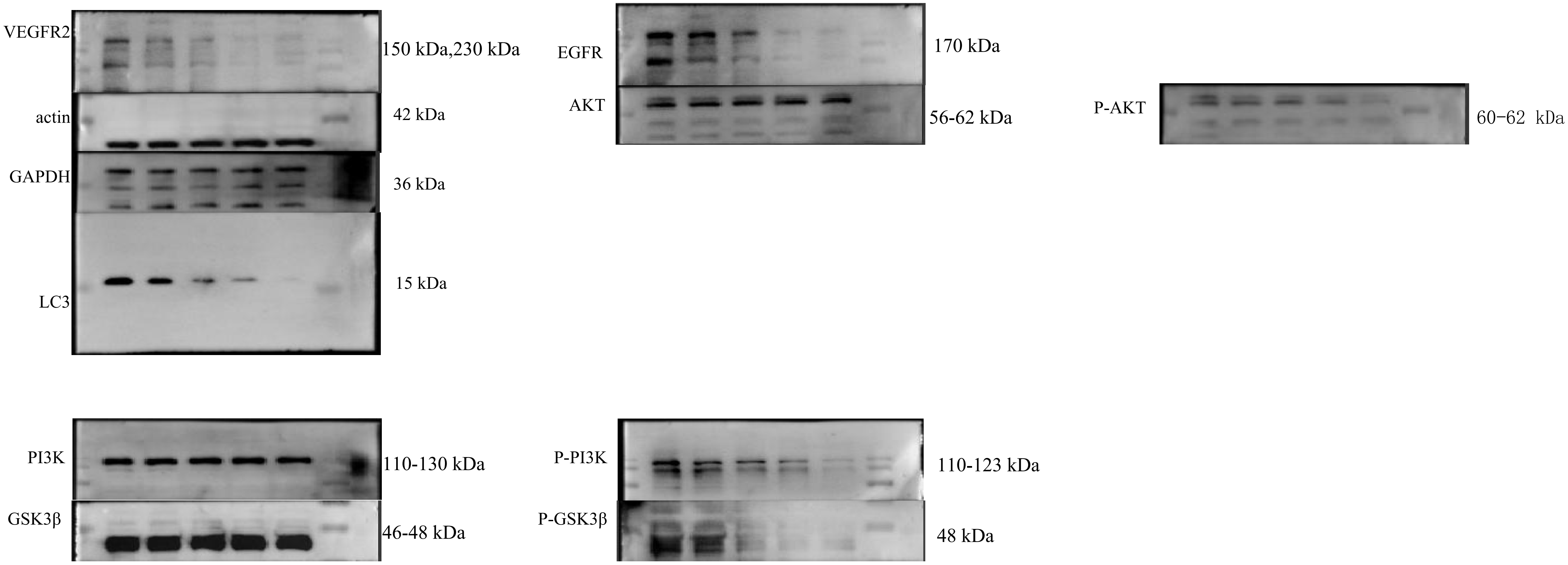

Figure 5B Huh-7-HBx

Repeat 3

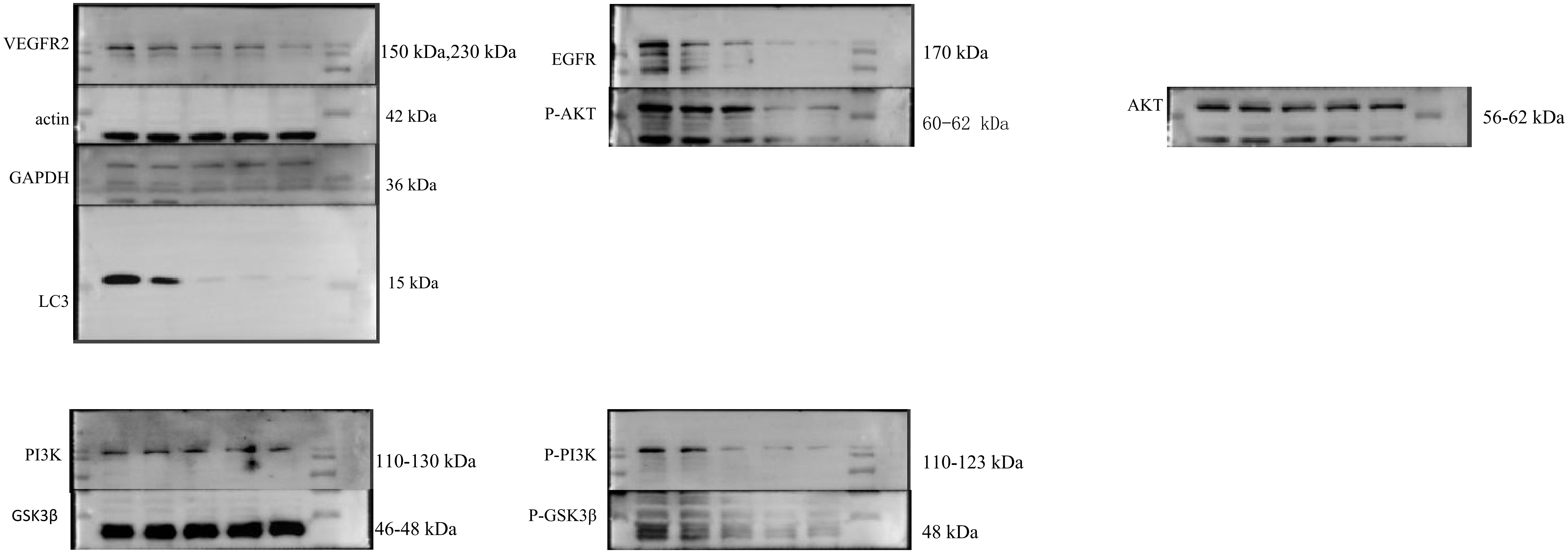

Figure 5C HepG2-HBx

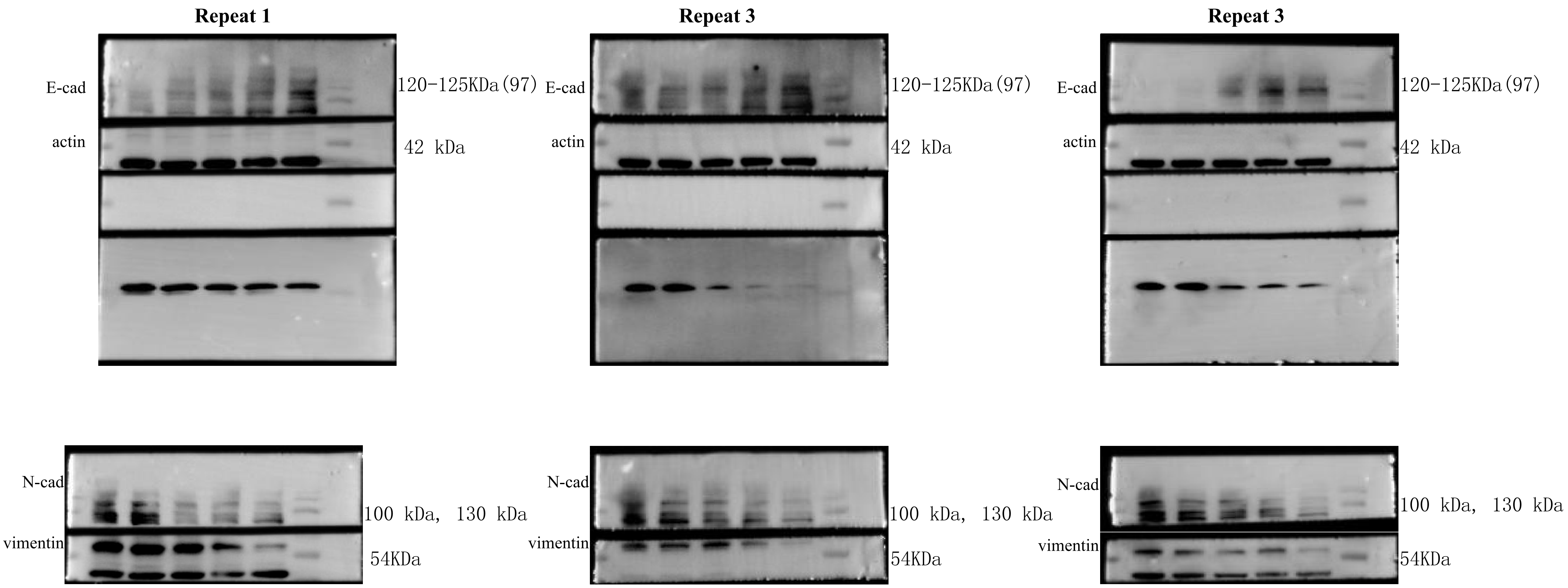

Figure 5D **Huh-7-HBx**

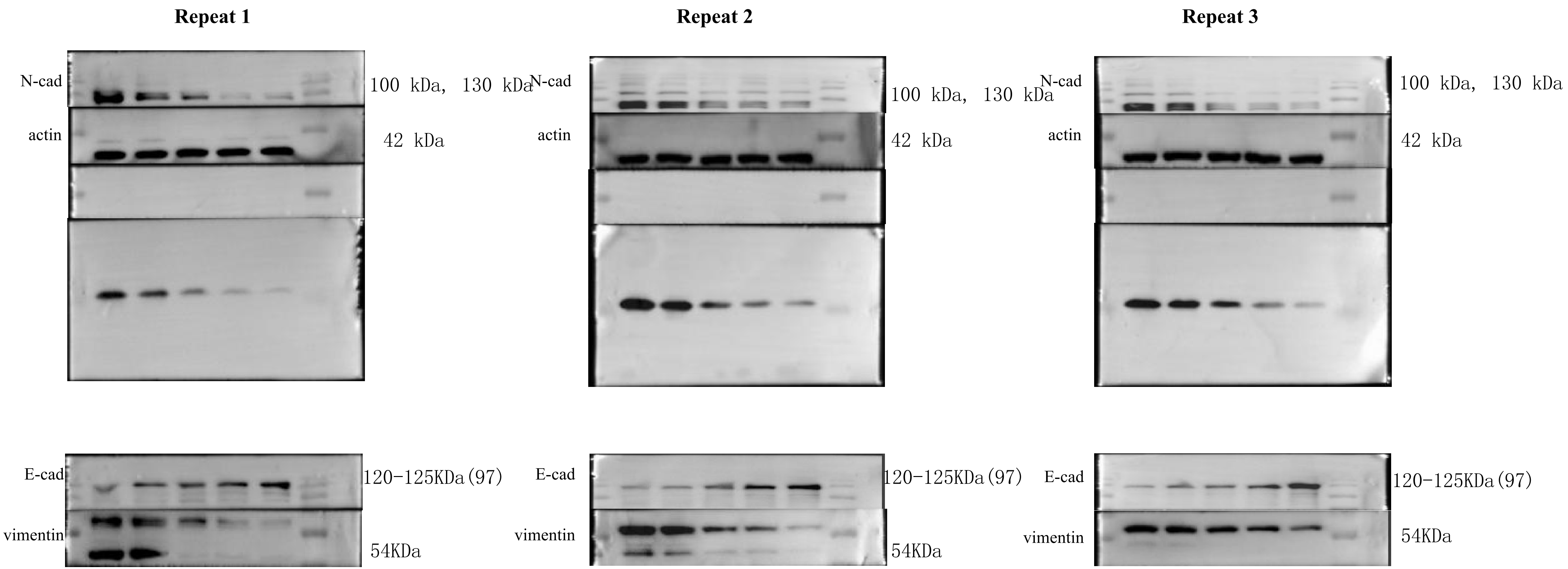

Figure 6A

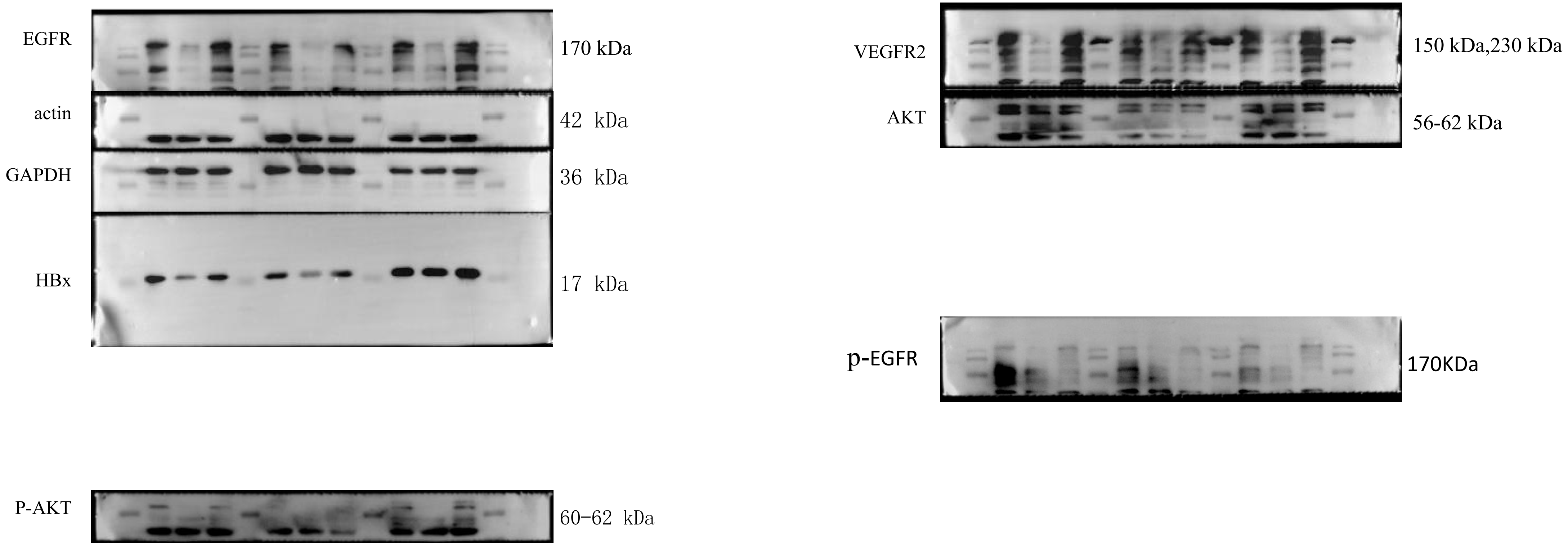
EGFR
170 kDa
actin
42 kDa
GAPDH
36 kDa
HBx
17 kDa
P-AKT
60-62 kDa
VEGFR2
150 kDa,230 kDa
AKT
56-62 kDa
p-EGFR
170KDa

Figure 6A

P-PI3K

actin

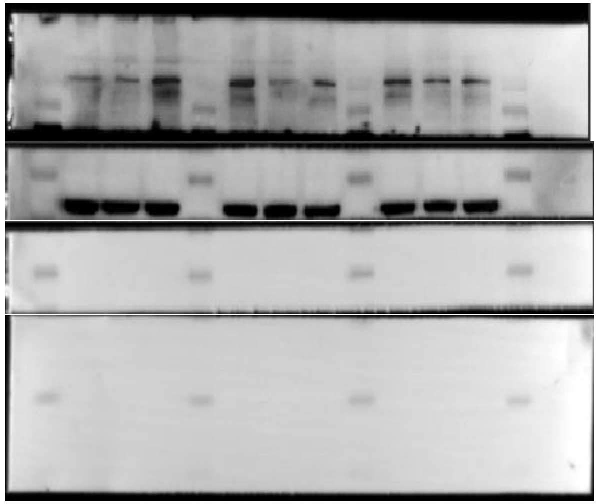

110-130 kDa

42 kDa

GSK3B

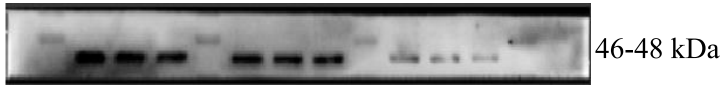

46-48 kDa

PI3K

P-GSK3B

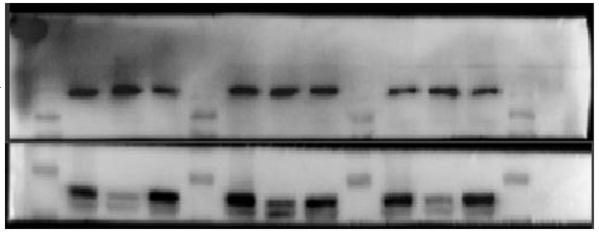

110-123 kDa

48 kDa

Figure 7F

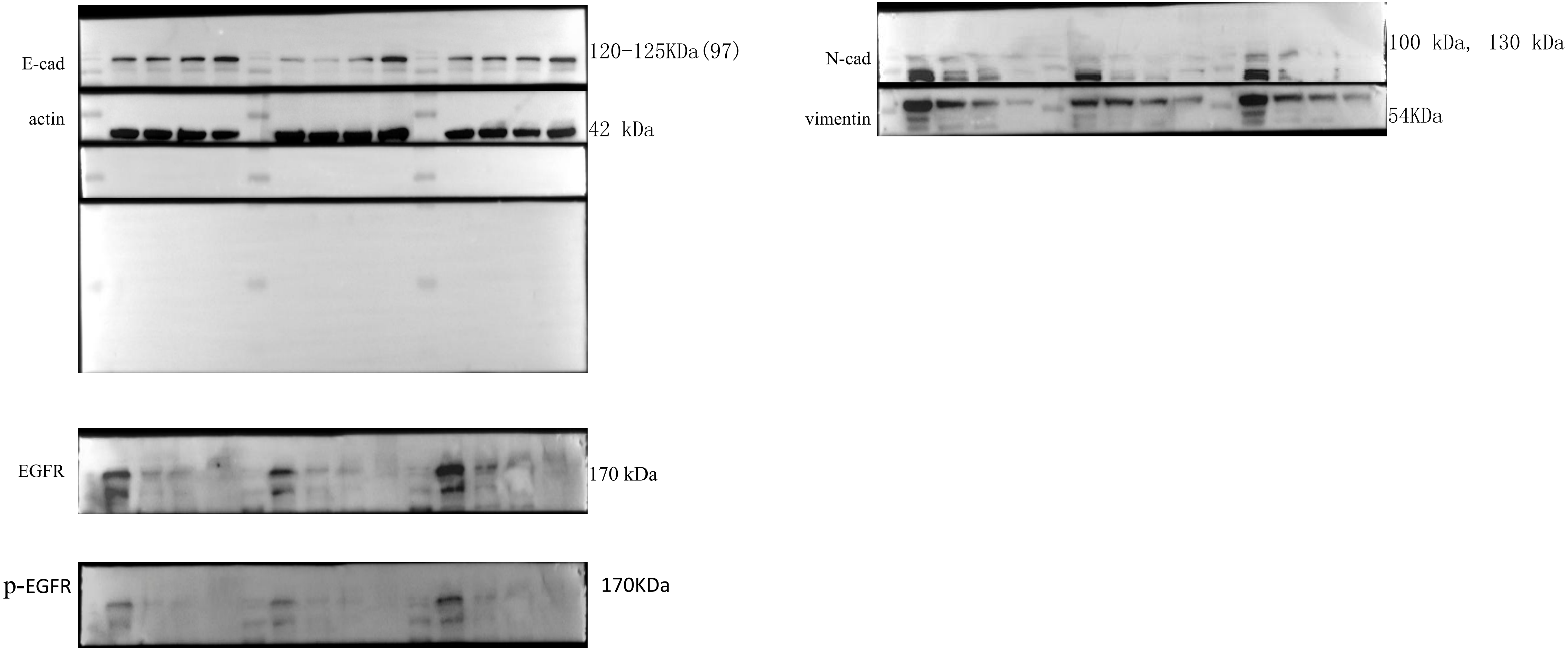
E-cad
120-125KDa(97)
actin
42 kDa
N-cad
100 kDa, 130 kDa
vimentin
54KDa
EGFR
170 kDa
p-EGFR
170KDa

S1 Figure F

EGFR 170 kDa

actin 42 kDa

GAPDH 36 kDa

HBx 17 kDa

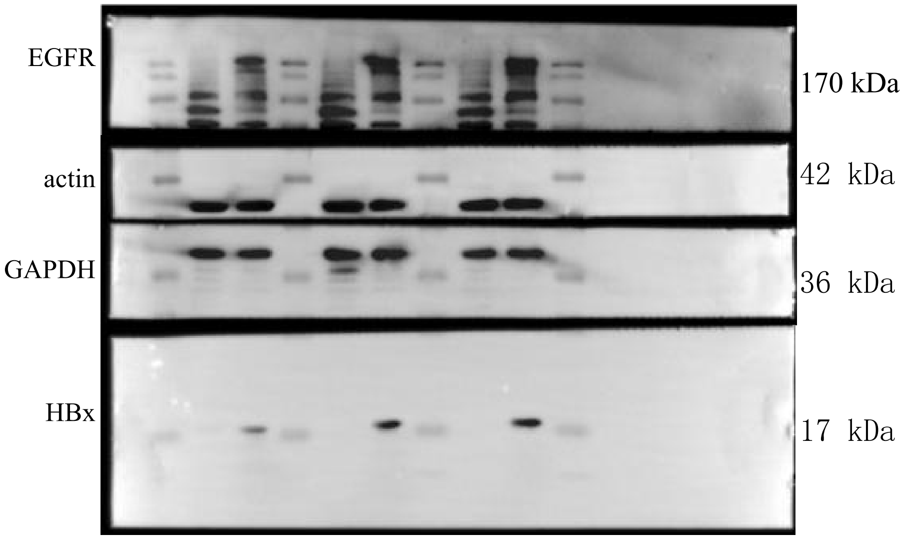

P-AKT 60-62 kDa

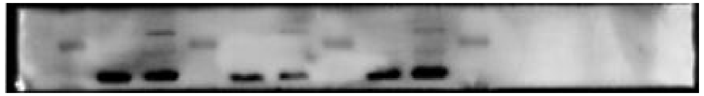

VEGFR 2 150 kDa,230 kDa

AKT 56-62 kDa

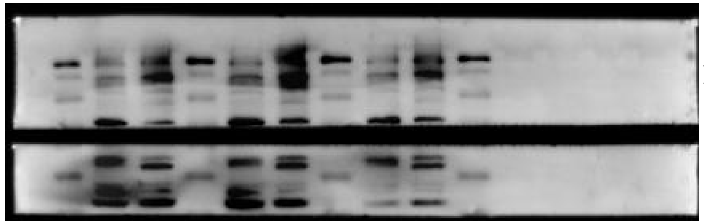

S1 Figure F

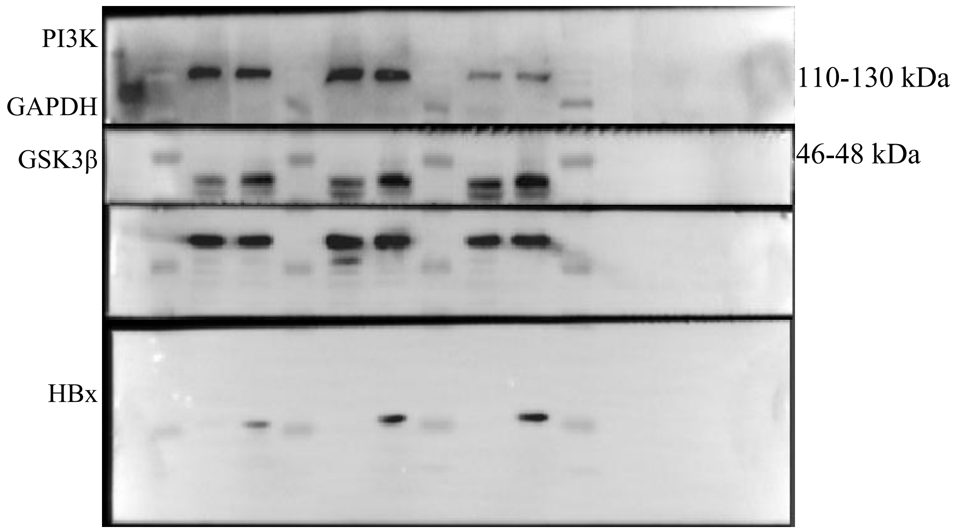
PI3K
GAPDH
GSK3β
HBx
110-130 kDa
46-48 kDa

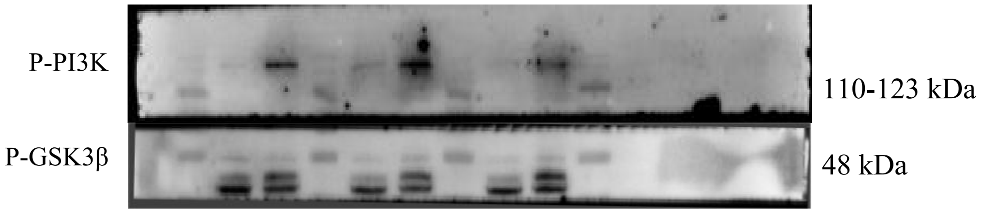
P-PI3K
P-GSK3β
110-123 kDa
48 kDa

S1 Figure G

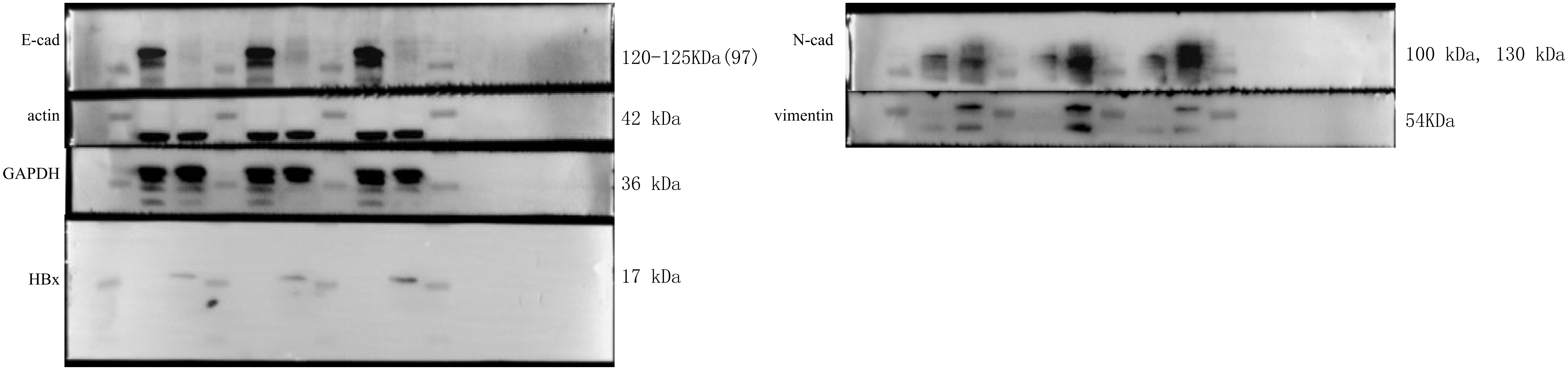
E-cad
120-125KDa(97)
actin
42 kDa
GAPDH
36 kDa
HBx
17 kDa
N-cad
100 kDa, 130 kDa
vimentin
54KDa

S1 Figure F, G   supplement

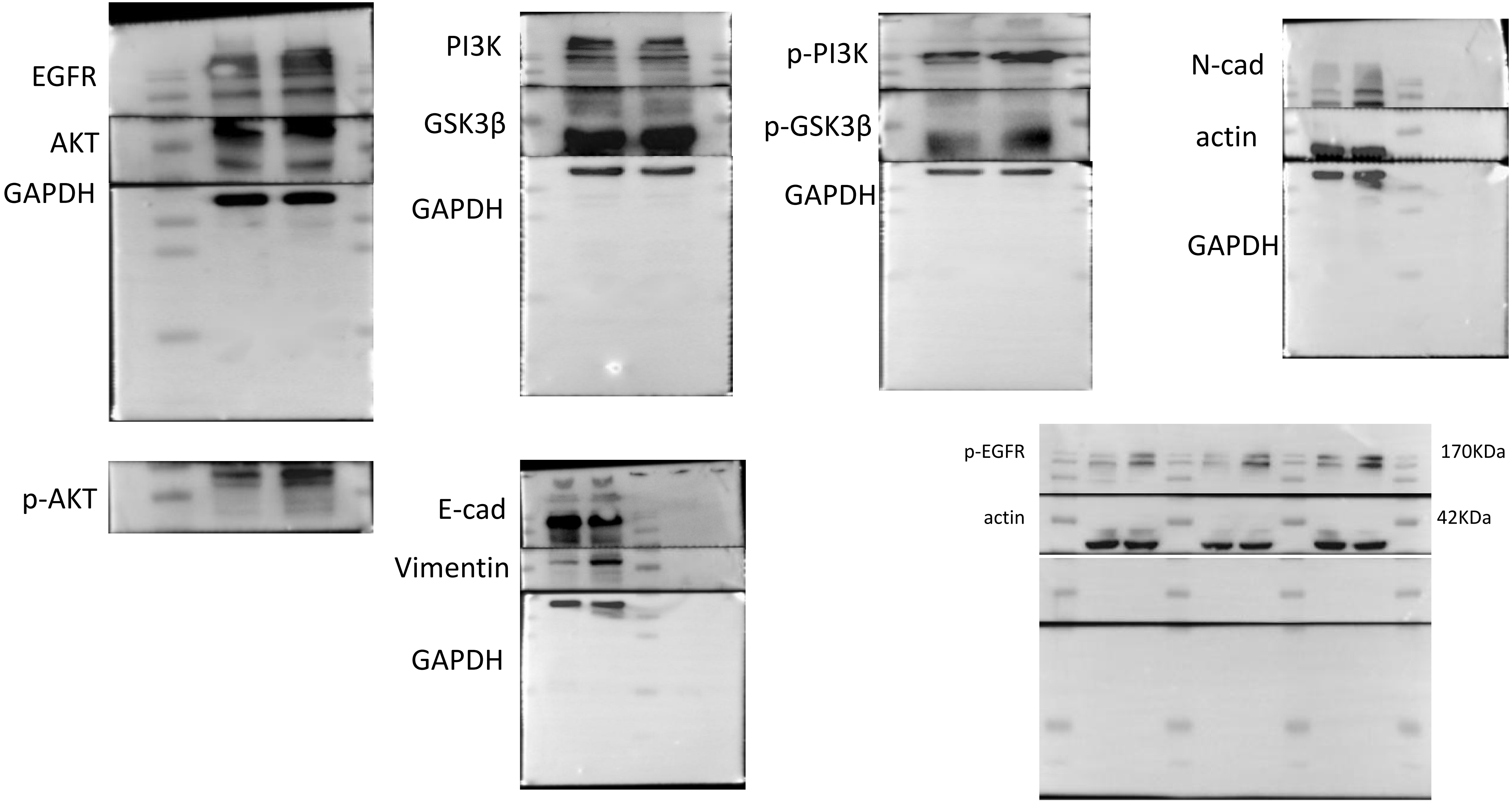

Supplement: S2 Fig — (PDF) [file pone.0350584.s004.pdf]
